# Hallmarks of lung cancer driven by inhalable particulate matter: From cell-intrinsic oncogenic traits to microenvironment remodeling

**DOI:** 10.1016/j.xinn.2026.101347

**Published:** 2026-03-18

**Authors:** Xiaoqiu Yuan, Xinrui Li, Chen Wang, Emilia Lim, Yunchu Wei, Ruoyi Jin, Lin Weng, Takahiro Karasaki, Hao Luo, Li Qing, Zhiwei Zhou, Qisi Jiang, Olga Chervova, Fan Yang, Fang Wu, Kai Zhang, Cheng-Yen Chang, Farrah Kheradmand, Jianpeng Sheng, David Carbone, Jun Wang, Kezhong Chen

**Affiliations:** 1 Department of Thoracic Surgery, Peking University People’s Hospital, Beijing 100044, China; 2 Thoracic Oncology Institute, Peking University People’s Hospital, Beijing 100044, China; 3 Research Unit of Intelligence Diagnosis and Treatment in Early Non-small Cell Lung Cancer (2021RU002), Chinese Academy of Medical Sciences, Peking University People’s Hospital, Beijing 100044, China; 4 Institute of Advanced Clinical Medicine, Peking University, Beijing 100191, China; 5 Peking University Health Science Center, Beijing 100191, China; 6 Department of Biochemistry and Molecular Biology, Faculty of Medicine, University of British Columbia, Vancouver, BC V6T 1Z3, Canada; 7 Edwin S.H. Leong Centre for Healthy Aging, University of British Columbia, Vancouver, BC V6T 1Z3, Canada; 8 Department of Thoracic Surgery, Graduate School of Medicine, The University of Tokyo, Tokyo 113-8654, Japan; 9 College of Bioengineering, Chongqing University, Chongqing 400044, China; 10 University of Texas Southwestern Medical Center, Dallas, TX 75390, USA; 11 School Hospital of Yangtze Normal University, Chongqing 408100, China; 12 University College London Cancer Institute, University College London, WC1E 6DD London, UK; 13 Department of Oncology, the Second Xiangya Hospital, Central South University, Changsha 410011, China; 14 Department of Environmental Health Sciences, School of Public Health, University at Albany, State University of New York, Rensselaer, NY 12144, USA; 15 Department of Medicine, Baylor College of Medicine, Houston, TX 77030, USA; 16 Center for Intelligent Oncology Designated by State Ministry of Education, Chongqing University, Chongqing 400030, China; 17 Chongqing Key Laboratory of Intelligent Oncology for Breast Cancer, Chongqing University Cancer Hospital and School of Medicine, Chongqing University, Chongqing 400030, China; 18 James Thoracic Oncology Center, Ohio State University, Columbus, OH 43210, USA

**Keywords:** lung cancer, particulate matter, tumor evolution, tumor microenvironment, air pollution

## Abstract

Inhalable particulate matter (PM) has emerged as a critical yet underappreciated carcinogenic factor in lung cancer, especially affecting non-smokers and populations residing in heavily polluted regions. Epidemiological research has confirmed the link between PM exposure and lung cancer risk, but the underlying biological pathways and clinical relevance are still not well understood. Here, we provide a comprehensive synthesis of current advances in PM-associated lung carcinogenesis, from population-level risk patterns to molecular pathogenesis and translational strategies. We outline the epidemiological burden and unique clinical features of PM-related lung cancer. We then propose a “Hallmarks of PM-associated lung cancer” framework, summarizing 12 features of how lung cancer evolves under PM exposure. This involves a complex interplay of cell-intrinsic oncogenic traits induced by PM (genetic alterations, epigenetic reprogramming, metabolic remodeling, stemness) and microenvironment reshaped by PM (inflammatory responses, immune suppression, angiogenesis, and pre-metastatic niche formation). Moreover, we critically examine the technical challenges in mechanistic studies and propose future solutions. Finally, we highlight urgent opportunities for integrating PM exposure into lung cancer screening, risk stratification, and prevention policies. This review offers a comprehensive framework for strategies to downsize the impact of air pollution on global lung health.

## Public summary

• Inhalable particulate matter (PM) is an established lung carcinogen, yet research remains globally uneven.
• We propose the “Twelve Hallmarks” framing PM-associated lung cancer from initiation through metastasis.
• PM drives epithelial oncogenic reprogramming via mutations, epigenetic dysregulation, and stemness.
• PM shapes microenvironment via inflammation, angiogenesis, and immunosuppressive/pre-metastatic niches.
• Hallmarks underpin distinct PM-lung cancer features, urging better models, biomarkers, and research equity.

## Graphical abstract

**Figure:**
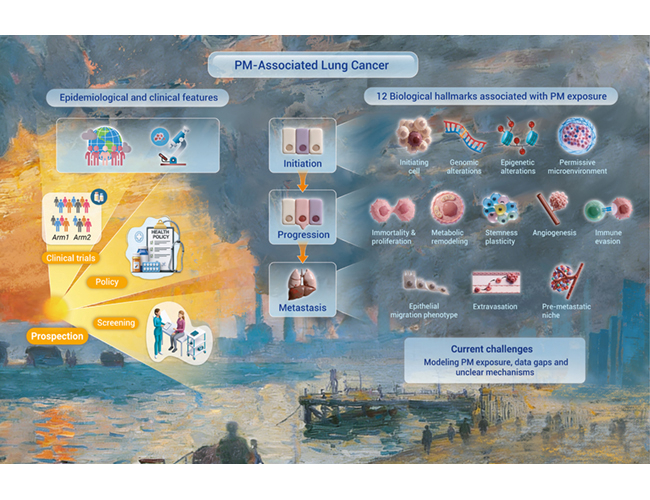

## Introduction

Lung cancer persists as the predominant source of global cancer-relevant mortality,^1^ with its rising incidence increasingly observed among non-smokers and individuals devoid of classical risk factors such as tobacco exposure.^2^^,^^3^^,^^4^ This shift has prompted growing attention toward environmental risk factors, notably inhalable particulate matter (PM), which the International Agency for Research on Cancer (IARC) has categorized as a Group 1 carcinogen.^5^ Growing epidemiological studies have consistently linked prolonged PM2.5 or PM10 exposure to a higher risk of lung cancer across diverse populations.^6^^,^^7^^,^^8^ According to limited available evidence, lung cancers arising under conditions of chronic PM exposure appear to exhibit distinct clinical and molecular features,^9^ underscoring the unique but poorly defined, carcinogenic pathway of PM-associated lung cancer.

The mechanisms through which PM contributes to lung carcinogenesis extend far beyond its initial toxicological categorization. Unlike classical mutagens that induce direct genotoxicity, PM is increasingly recognized as a chronic environmental stressor capable of launching complex biological processes. These may include not only DNA damage^10^ but also epigenetic dysregulation, prolonged inflammation, immune suppression, and remodeling of the lung microenvironment,^11^^,^^12^ most of which remain incompletely elucidated. Additionally, how PM orchestrates different facets of pathways to drive the stepwise evolution of lung cancer remains largely unsolved. Meanwhile, major limitations persist in experimental research. Conventional *in vitro* and *in vivo* models frequently fail to recapitulate the chronic, low-dose, and compositionally complex nature of real-world PM exposure, restricting the translation of mechanistic findings into pragmatic strategies.^13^^,^^14^

To address these gaps, our review first traces the spatiotemporal trajectory of PM research and its striking global disparities, setting the stage for a detailed examination of how the physicochemical properties of PM shape its carcinogenic potential. Guided by this foundation, we then manage to pursue a deeper understanding of PM-relevant lung cancer through three lines of inquiry. We start with establishing the unique clinical and epidemiological profiles of this disease entity, followed by deciphering the underlying biological mechanisms, which formalize in the novel framework of twelve biological hallmarks for carcinogenesis under PM exposure. While PM involves a range of particles with diverse sizes and compositions, PM2.5 emerges to contribute predominantly to lung cancer^15^^,^^16^ and most previous non-human experimental studies have focused on PM2.5, rendering it the major focus of our mechanistic analysis. We also incorporate insights from studies on PM10 and other significant subcategories to present a comprehensive view. Finally, we chart a course for translating these insights into clinical and public health practices. By synthesizing evidence across these four domains, this review aims to provide a comprehensive roadmap for understanding, detecting, and ultimately mitigating the global burden of PM-associated lung cancer.

## Spatiotemporal trajectory and global disparities in PM and lung cancer

**Figure 1:**
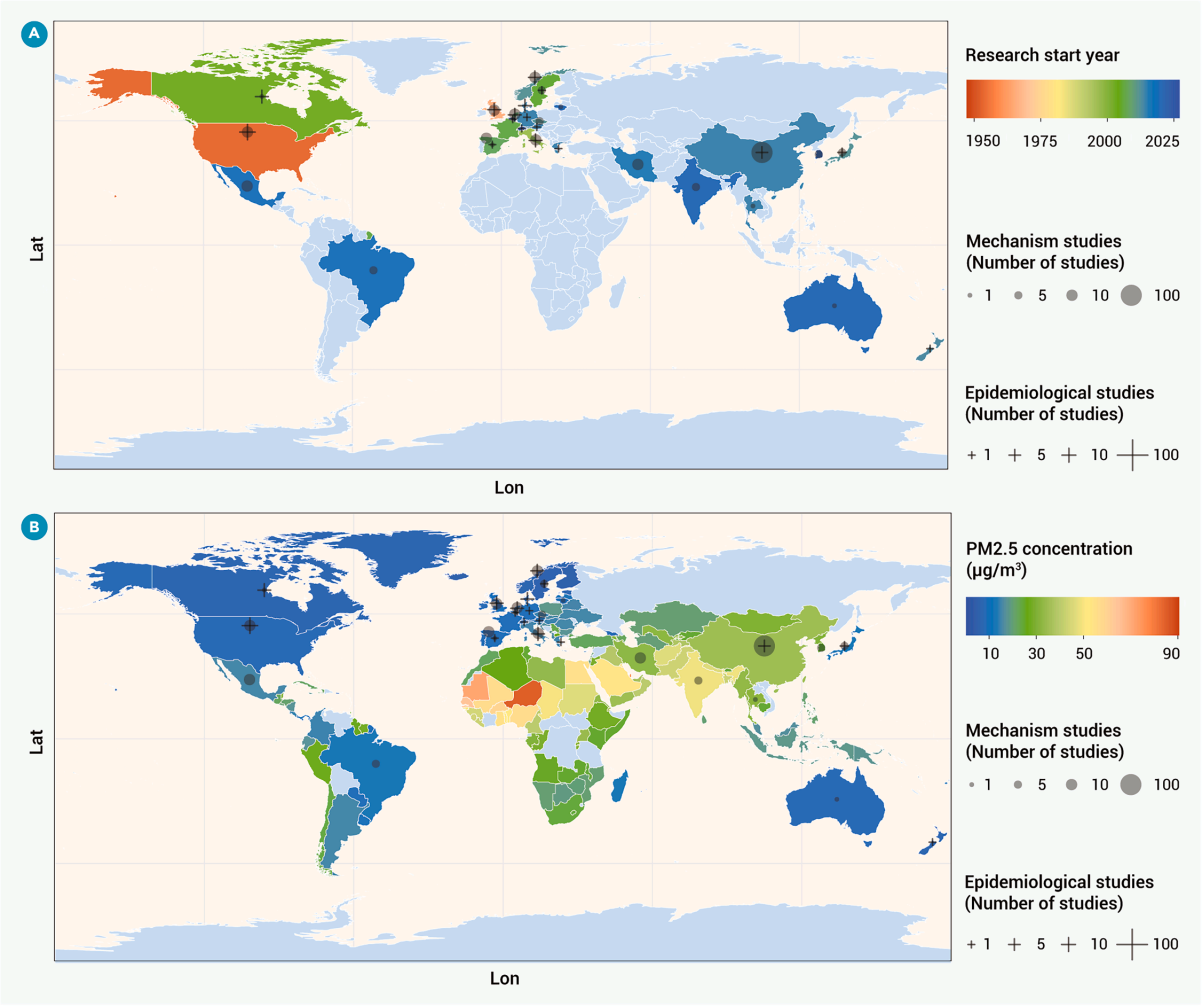
Global distribution of research studies and PM2.5 concentration levels (A) This figure illustrates the starting time of high-quality PM research in different countries or regions, indicating their potential for early foundational work and sustainable leading studies, compared in relation to pollution levels shown in (B). Geographic distribution of the earliest high-quality studies (including both epidemiological and mechanism studies, and several pioneering review studies) on PM2.5 exposure. The color gradient indicates the year of earliest high-quality studies in each country or region, ranging from earlier years (1990) in red to more recent years (2025) in blue. The size of gray circles represents the number of mechanism studies (Table S3) conducted in each country or region, while gray crosses indicate the number of epidemiological studies (Table S1). (B) This map illustrates the global distribution of population-weighted PM2.5 pollution, measured in micrograms per cubic meter (μg/m^3^), on a gradient scale from low (blue, 10 μg/m^3^) to high levels (red, 90 μg/m^3^). Air pollution data are overlaid with the number of country (or region)-level publications on PM2.5 from Table S1 (epidemiological studies) and Table S3 (mechanism studies). #Air pollution data were obtained from the Global Burden of Disease (GBD) database. Expected exposure concentrations were averaged over the 10 years preceding the final date of the study’s screening year. For studies with the screening year earlier than 2000, data from as early as 1990 were used. ##A rigorous procedure was used to filter high-quality studies. We searched the PubMed and Web of Science databases using the following terms: (lung cancer/lung adenocarcinoma/lung squamous cell carcinoma [title/abstract]) AND (air pollution/air pollutant/particulate matter [title/abstract]) AND (“1990/01/01”[Date - Publication]: “3000”[Date - Publication]). Inclusion and exclusion criteria for epidemiological studies: Inclusion criteria: studies that reported specific hazard ratios (HR) or odds ratios (OR) for incremental increases in PM. Exclusion criteria: studies with small sample sizes (approximately fewer than 1,000 participants) or retrospective cohort studies were excluded, except in cases where the study demonstrated groundbreaking significance. Inclusion criteria for mechanism studies: Studies directly related to PM or those indirectly reflecting the effects of certain PM components (e.g., PAHs). These studies were systematically summarized in Table S1 (epidemiological studies) and Table S3 (mechanism studies). ###The country or region where the mechanism studies were conducted is defined based on the geographic location of the first author’s affiliated institution. ####The country or region where the epidemiological studies were conducted is defined based on the geographic location(s) of the cohort population adopted from. #####For global overview of PM2.5 pollution levels and research distribution in 2020 and other years (1990–2020), see Figures S1–S3.

The link between air pollution and lung cancer has captured researchers’ interest since at least the 1950s (Figure 1). While the influence of environmental factors on lung diseases was acknowledged, research primarily concentrated on occupational exposures, leaving the effects of inhalable PM insufficiently studied.^17^^,^^18^ Robust epidemiological evidence began to emerge in the late 20th century. The landmark Harvard Six Cities Study first reported that prolonged exposure to low concentrations of PM was linked to a heightened mortality risk associated with lung cancer^19^ and was later corroborated by the long-term follow-up (1982–1998) of the American Cancer Society (ACS) II cohort,^20^^,^^21^ which involved nearly half a million adults from urban regions in the United States. Recent global-level measures further strengthened the epidemiological foundation. The relative risk of lung cancer across 204 countries was observed to continuously rise with increasing levels of ambient and household PM exposure. Unlike ischemic cardiovascular diseases or chronic obstructive pulmonary diseases, the risk curve for lung cancer demonstrates no apparent threshold or plateau effect throughout the measured concentrations of 0–600 μg/m^3^.^22^ These findings prompt the World Health Organization (WHO) to establish air quality guidelines,^23^ and render the carcinogenic mechanisms of inhalable PM to be a critical research focus. By the turn of the 21st century, with tobacco under control and the elevating proportion of non-smoking lung cancer, research on PM-related lung cancer has evolved into a distinct scientific domain. Inhalable PM extracts or industrial standards have been used in preclinical settings to investigate responsive biological effects, revealing DNA damage and immune modulation.^24^^,^^25^ Over the past two decades, investigators have progressively constructed a mechanistic map detailing the relationship between air pollution and lung cancer (see later sections).

Despite these advances, striking geographic disparities persist. The global distribution (Figure 1) exhibits widespread mismatching between PM2.5 exposure burdens and the maturity or breadth of corresponding etiological investigations. Higher PM2.5 levels and lung cancer mortality mainly occur in low- and middle-income regions (South Asia, Southeast Asia, and Africa). This imbalance persists even in meta-analyses that adjust for smoking,^8^ where air pollution-attributable, age-standardized disability-adjusted life-year (DALY) rates are highest in South Asia, sub-Saharan Africa, and parts of North Africa and the Middle East. However, the majority of mechanistic and epidemiological studies involving PM-relevant lung cancer were conducted in high-income regions with lower pollution such as North America and Europe. This imbalance exacerbates the knowledge gaps in highly exposed regions.^26^ Disproportionate research efforts necessitate equitable scientific and policy attention to the regions most affected by air pollution.

## The role of PM properties and categories

Before vertically discussing how PM affects lung cancer, it is necessary to horizontally clarify the nomenclatures of PM that have appeared in previous studies, which were mostly termed by the intrinsic properties and related categories. The carcinogenicity of PM cannot be understood without considering these elements. Particle size determines the depth of deposition within the respiratory tract, whereas composition shapes its molecular reactivity and biological potency. Together, these factors shape the distinct toxicological profiles of various PM subtypes and sources.

### Influence from inherent physical and chemical characteristics of PM

PM is commonly classified by aerodynamic diameter into coarse particles (PM10), fine (PM2.5), PM1, and ultrafine particles (UFP, PM0.1),^27^ each dictating distinct deposition patterns within the respiratory tract.^28^^,^^29^ While *in vitro* experiments revealed similar activated pathways for PM2.5 and PM10, such as elevating interleukin-6 (IL-6) levels^30^^,^^31^ and promoting epithelial-to-mesenchymal transition (EMT),^16^^,^^30^ the *in vivo* processing of these particles in the lungs resulted in biological differences. PM10 are primarily deposited in the upper airways and are effectively cleared by mucociliary action, resulting in more localized effects.^32^ In contrast, PM2.5 and smaller particles penetrate deeply into the distal lungs, reaching the small bronchi and alveoli.^27^ This permits extensive interaction with the lung epithelium and potential translocation into systemic circulation, thereby amplifying their toxicity.^33^ Notably, PM2.5 possesses a high surface area-to-mass ratio, which facilitates the adsorption of toxic compounds such as polycyclic aromatic hydrocarbons (PAHs), rendering it more toxic than PM10.^15^^,^^16^ Although PM1 and UFP tend to exhibit even higher toxicity than PM2.5, relating to the enrichment of carbonaceous elements and hazardous metals,^34^ they remain underexplored on carcinogenesis without high-quality evidence. In contrast, PM2.5 has been dominantly explored and found to induce significantly higher rates of cell migration and invasion than PM10 at equivalent concentrations.^16^ Consequently, due to these potent physicochemical properties and its stronger epidemiological links to adverse cardiorespiratory outcomes,^15^ PM2.5 has become the primary focus of mechanistic and public health studies.

Chemical composition is another critical determinant of biological effects. PM is a complex mixture that includes inorganic ions (sulfate, nitrate, ammonium), elemental carbon, organic compounds, and a range of transition metals (nickel, cadmium, chromium).^27^ Among these components, PAHs are notable for their strong carcinogenic potential,^35^ driven by their capacity to induce DNA damage, adduct formation, and genomic instability in respiratory cells.^36^ Studies focusing on extracted PAHs also revealed the activation of tumor-promoting pathways such as oxidative stress and EMT in A549 cells,^37^ especially the high-molecular weight (HMW) PAHs.^38^ Although transition metals exhibit less toxicity than PAHs in normal human bronchial epithelial cell line BEAS-2B,^39^ they can catalyze ROS formation and proline metabolism^39^ exacerbate DNA impairment,^40^ and trigger adaptive antioxidation mediated by Nrf2 which facilitates resistance to apoptosis^41^ in human bronchial epithelial and lung adenocarcinoma (LUAD) cell lines. Epidemiologically, PM components of sulfur and nickel^42^ were reported associated with lung cancer risk,^43^^,^^44^^,^^45^ which have been classified as carcinogenic to the lungs by IARC.^46^^,^^47^

The specific mixture of PM varies with particle size, emission source, combustion conditions, and environmental climates^48^^,^^49^^,^^50^ which shapes the observed biological results. For example, larger particles (PM10) mainly contain crustal material and microbial materials, while finer particles are dominant by heavy metals and organic combustion products such as PAHs, aligning with the greater carcinogenic capacity of PM2.5 and PM0.1 in normal human bronchial epithelial cells isolated from airway biopsy.^51^ As for automobile exhaust, the fuel type and working mode of engines largely affect the PM constituents. Diesel engines generate significantly higher amounts of PAHs than gasoline ones in A549, with the idling operation of engine producing even more PAHs than high-speed mode.^52^ Concerning geographical distinctions, urban PM2.5 induces more severe genotoxicity than rural and industrial PM2.5 in BEAS-2B,^53^ partially relevant to its higher abundance of PAHs as well as metals.^54^ Within urban PM, winter PM2.5 causes higher biotoxicity than summer in A549 cells.^55^

### Physicochemical and toxicological profiles of indoor PM

Indoor air pollution has received relatively limited attention in previous studies and disproportionately affects populations in developing or underdeveloped countries.^56^ Most large cohorts linking PM exposure to lung cancer risk rely on outdoor measurements derived from fixed-site monitoring or satellite-based models. Published mechanistic studies also predominantly utilized reference materials or collected samples representing ambient outdoor PM2.5 or PM10. Thus, the epidemiological associations and molecular pathways summarized in this review largely reflect ambient exposure. However, it is important to acknowledge that indoor PM constitutes a major contributor to personal exposure, particularly aerosols generated from frying, stir-frying, smoking oils, and biomass cooking.

Indoor PM differs from outdoor PM in its physicochemical profile, typically containing higher levels of ultrafine particles, organic aerosols, aldehydes, and lipid oxidation products.^38^ Although the patterns of direct biological effects caused by indoor PM2.5 align closely with those of ambient PM2.5, which converges on oxidative stress and chronic inflammation, indoor PM is capable to trigger stronger response in A549, further observed in the airways of those chronically cooking with biomass fuel.^57^^,^^58^ Specific indoor combustion sources further introduce distinct toxicological profiles. For instance, environmental tobacco smoke (ETS) and incense smoke (IS) are contributors to indoor PM2.5 pollution. ETS has a higher PM2.5 emission factor compared with IS, while IS emits greater amounts of PAHs and carbonyl compounds. ETS demonstrates stronger potential for inducing oxidative stress in A549 cells. In contrast, IS elicits a more pronounced inflammatory response.^38^ Another notable contributor to indoor air pollution is indoor dust, which contains toxic components such as extracellular vesicles (EVs). These EVs are linked to pulmonary inflammation and may promote lung metastasis by inducing TNF-α and facilitating neutrophil migration.^59^ In regions frequently using household coal combustion such as Xuanwei of China, benzo[*a*]pyrene (BaP) has been identified by proteogenomic analysis as a primary component of indoor PM, which strongly associates with the unique mutation patterns of lung cancer patients in these places.^60^

### Comparison among PM with different categories

**Figure 2:**
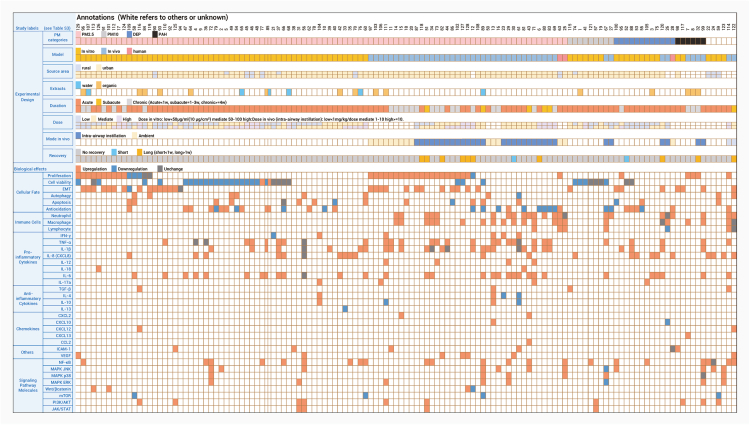
Observed biological effects of PM on lungs and the impact of experimental details The biological impact of different PM exposure scenarios as examined in previous studies were illustrated, alongside the selected models utilized in each experiment. Each column of the heatmap represents a reported experimental study, and the top number is the study number, which corresponds to Table S3. Also, each row represents an experimental mode or an observed experimental phenomenon category. For each row, different colors are used to represent different experimental processing settings. The square containing two rectangular splicing indicates that two experimental condition modes are adopted in this study. This figure was arranged based on changes in proliferation, cell viability, other pro-tumor phenotypes, as well as doses and duration of PM exposure. A comprehensive analysis is provided on the alterations observed in key cytokines, pathways, cellular events, and the recruitment of immune cells across the study findings. See Table S3 for details of each study.

As summarized in Figure 2, where each row represents one biological response in previous studies, PM of different categories don’t show vast heterogeneity, but produce similar patterns of molecular outcomes on lung cells, either consistently promoting or inhibiting. Certain unique characteristics were also noticed for specific PM subtypes. Compared with PM2.5 or PM10 directly collected from the atmosphere, the impact of diesel exhaust particles (DEP) is often more moderate in cytotoxicity but more cancer-fostering. For example, studies have shown that DEP induces significant cytotoxicity in multicellular epithelial tissue barrier models only at higher concentrations and prolonged exposure durations than those of PM2.5.^61^^,^^62^ Rather, it inhibits serum starvation-induced apoptosis of A549^63^ and promotes metastasis via neutrophilic lung inflammation in female mice.^64^ Furthermore, in contrast to PM2.5, which increases CXCL10 expression^65^ and promotes T cell recruitment (especially Th1 cells),^66^^,^^67^ DEP suppresses Th1 signaling via aryl hydrocarbon receptor (AHR)-mediated pathways^68^ both *in vitro* and *in vivo*. DEP exposure appeared associated with a shift toward Th2-type immune responses.

Although Figure 2 adopted the PM nomenclature employed in the original study as categories, it is essential to recognize that DEP, indoor PM, ambient PM2.5 and PM10 are not mutually exclusive entities. Rather, they represent classifications with substantial overlap. For instance, DEP, defined by its source, largely falls within the PM2.5 size fraction, while a major portion of indoor PM, derived from cooking and combustion, also belongs to PM2.5. In fact, toxicological studies on DEP or specific indoor PM sources reveal the biological impacts of PM2.5 subpopulations with distinct chemical compositions.^56^^,^^69^^,^^70^ Therefore, in most subsequent discussions, we did not address each category separately but instead concentrated on the convergent mechanisms of inhalable PM, especially the PM2.5.

## Defining lung cancer associated with PM

Based on the physicochemical properties and classifications of PM, we can now delineate how these characteristics manifest in the distinct epidemiological, clinical, radiological, and molecular features that define PM-associated lung cancer as a unique disease entity.

### Epidemiological risks of PM-associated lung cancer

Inhalable PM has consistently increased lung cancer risk across diverse regions, exposure contexts, and populations (Table S1). Multiple cohort studies across Europe, North America, and Asia have reported positive exposure-response relationships for PM2.5, with hazard ratios (HRs) ranging from approximately 1.07 to 1.16 for every 10 μg/m^3^ rise in PM2.5 levels.^71^^,^^72^^,^^73^^,^^74^^,^^75^^,^^76^^,^^77^ However, the shape of this relationship appears to be modified by the prevailing exposure level. At low to moderate PM2.5 concentrations (<30 μg/m^3^), the exposure-response curve tends to be near-linear. In regions with heavy pollution (PM2.5 ≥ 35 μg/m^3^), the risk curve deviates from linearity, exhibiting either a plateau effect^78^^,^^79^ or a superlinear acceleration.^80^ As for outcomes specific to lung cancer, earlier studies (1992–2015) have reported positive U-shapes for the curve between PM2.5 concentration and HRs of lung cancer mortality,^80^ while more recent studies (2013–2017)^78^ have yielded inverted U-shapes, with inflection points observed at approximately 60 μg/m^3^. A similar saturation effect at around 75 μg/m^3^ for PM2.5 has been noted in a global analysis of all-cause mortality,^15^ suggesting a broader pattern of attenuated risk increase at extreme exposure levels. We will discuss the biological ground underlying the non-linear patterns in later section.

PM10 has also been linked to adverse lung cancer mortality, with HRs ranging from 1.05 to 1.50 per 10 μg/m^3^ increment.^45^^,^^81^^,^^82^^,^^83^^,^^84^^,^^85^^,^^86^ Concerning limited evidence, similar exposure-response curves were observed for PM10 compared with PM2.5, with higher turning concentration at around 150 μg/m^3^.^15^ Research on UFPs remains inconclusive mainly due to methodological inconsistencies and limited longitudinal data. Some suggest modest associations with lung cancer mortality,^87^^,^^88^^,^^89^ whereas others show no significant correlation.^90^ Studies focusing on specific PM sources, such as DEP, indicate a robust association with lung cancer across smoking statuses.^91^^,^^92^^,^^93^^,^^94^^,^^95^ Risks posed by inhalable PM of other origins require further elucidation, including forest fires, coal-burning power plants, and industrial activities.^96^^,^^97^

Apart from measurements at the population level, PM exposure also worsens individual clinical outcomes of lung cancer.^98^^,^^99^^,^^100^^,^ Higher PM levels significantly shorten the patient survival, especially in localized or regional-stage disease.^101^ Similar findings have been reported regarding post-operative exposure^102^ and acute PM2.5 spikes.^103^^,^^104^ On the other hand, improvements in ambient air quality may offer measurable prognostic benefits,^105^ underscoring the potential impact of public health interventions beyond primary prevention.

### Heterogeneity of epidemiological associations across subgroups

The carcinogenic effect of PM exposure is not uniformly distributed across all populations, and susceptibility may vary based on demographic, behavioral, and genetic factors. Age is a notable modifier, with older individuals generally exhibiting higher vulnerability to PM-related lung cancer.^80^^,^^106^ Studies both in low and high-pollution areas^107^^,^^108^ have demonstrated more evident risk associations in those older than 65 years^109^ In the ESCAPE study, the HR value of lung cancer linked to a 10 μg/m^3^ rise in PM2.5 levels was 1.04 for those under 65 and 1.38 for those above 65 years old.^42^

Sex-specific differences remain inconclusive,^71^^,^^77^ with existing studies offering mixed results.^110^^,^^111^^,^^112^ While the Nurses’ Health Study reported a modest increase in risk for PM2.5,^112^ cohorts focusing on men showed similar results to the general population.^113^ However, few analyses have adequately adjusted for indoor air pollution, which disproportionately affects women in certain cultural contexts due to cooking practices or fuel use.^114^

Concerning the smoking status, heightened risks have been identified in both never-smokers and ever-smokers under PM exposure,^42^^,^^71^^,^^73^^,^^76^^,^^77^^,^^112^ with disproportionately elevated relative risk in non-smokes.^80^^,^^108^ On the other hand, air pollution and tobacco smoke can act synergistically to exacerbate lung carcinogenesis, partially contributing to excess mortality beyond additive expectations for smokers.^93^^,^^115^^,^^116^

Genetic predisposition further modulates individual susceptibility to PM. In UK Biobank, individuals with high polygenic scores exhibit amplified lung cancer risk by 37% when exposed to elevated PM2.5 concentrations.^117^ Similarly, individuals with significant PM2.5 exposure and elevated genetic risk exhibit the greatest vulnerability to lung cancer.^118^^,^^119^^,^^120^ These data advocate for more refined prediction models that incorporate PM exposure along with demographic as well as genetic covariates to identify at-risk individuals who fall outside the scope of traditional screening paradigms.^121^

### Phenotypical features of PM-associated lung cancer

**Figure 3:**
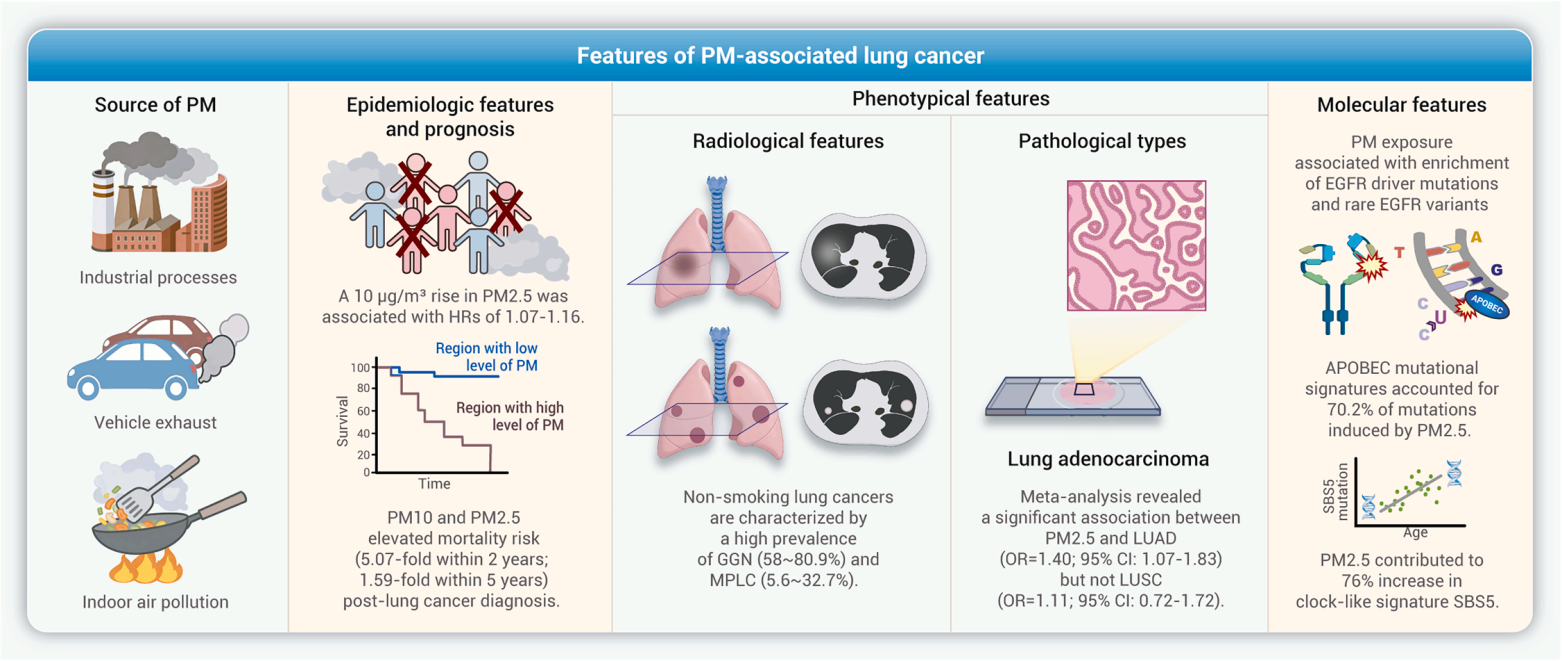
Features of PM-associated lung cancer This illustration summarizes the reported features of lung cancer associated with PM, encompassing its sources of carcinogens, epidemiological and clinical characteristics, and molecular alterations. The primary sources of PM include industrial processes, vehicle exhaust, and indoor air pollution. Epidemiological studies have demonstrated elevated lung cancer incidence and mortality rates in populations exposed to high PM, accompanied by poor prognosis compared with non-PM-exposed cases. Radiological features of PM-associated lung cancer often include ground-glass nodules (GGNs) and multiple primary lung cancers (MPLCs), predominantly observed in non-smoking populations. Pathologically, lung adenocarcinoma is the dominant subtype linked to PM exposure. At the molecular level, PM exposure is associated with driver mutations in EGFR and mutational signatures catalyzed by APOBEC enzymes (C→U transitions). These findings highlight the distinct features of PM-associated lung cancer, providing insights that could guide clinical applications and inform targeted research efforts. PM, particulate matter; EGFR, epidermal growth factor receptor; APOBEC, apolipoprotein B mRNA editing enzyme, catalytic polypeptide-like.

Lung cancers arising from prolonged PM exposure, often observed in non-smokers within East Asian populations, show unique clinical and molecular characteristics (Figure 3). Radiologically, these patients tend to more frequently present with ground-glass nodules (GGNs). A notably high prevalence of multifocal disease, including multiple primary lung cancers (MPLCs), has also been reported in these settings (Table 1). For instance, a province-wide lung cancer screening trial involving non-smokers in Taiwan (China) reported that over 80% of positive cases exhibited GGNs, including 48.0% pure GGNs and 32.9% part-solid nodules, with 17.9% presenting with MPLCs.^123^ These imaging patterns differ from the solid and solitary nodules commonly seen in traditionally smoking-related lung cancers,^131^ potentially indicating an alternative pathway for tumor onset and progression.

**Table 1:** Radiological features of non-smoking lung cancers in screening trials in areas with heavy air pollution

CT features data for non-smokers	Author (year)	Participants	GGN nodules in non-smoking lung cancer	MPLC patients in non-smoking lung cancer	Estimating population-weighted PM concentrations in the study population (μg/m^3^)
Reported	Kang et al.^122^ (2018)	Korea	80.8% (42/52), pGGN: 32.7% (17/52), PSN: 48.1% (25/52)	32.7% (17/52) display multiple lung nodules	25.15
	Chang et al.^123^ (2024)	Taiwan, China	80.9% (327/404), pGGN: 48.0% (194/404), PSN: 32.9% (133/404)	17.9% (57/318)	21.87
Not reported	Yi et al.^124^ (2015)	Korea	low-risk by smoking history and age, 68.3% (41/60), pGGN: 43.3% (26/60), PSN: 25.0% (15/60)	NA	24.84
	Li^125^ (2021)	Tianjin, China *N* = 5,523 (68.2% non-smokers)	low-risk by smoking history and age, 73.7% (14/19), pGGN: 26.3% (5/19), PSN: 47.4% (9/19)	NA	49.15
	Zhang et al.^126^ (2020)	Mainland, China	93.3% (167/179) non-smoking lung cancer, GGN: 96% (171/179) in all, NA about pGGN or PSN	NA	49.98
	Wu et al.^127^ (2016)	Taiwan, China	92% (23/25) non-smoking lung cancer, GGN: 93.8% (30/32) in all nodules, pGGN: 43.8% (14/32) in all, PSN: 50% (16/32) in all	28% (7/25) of all lung cancer patients (92% non-smokers) 5 pGGN+PSN 2 both pGGN	22.67
	Liu et al.^128^ (2011) 03-09 screen	China	65% (22/34) non-smoking lung cancer, GGN: 58% (21/36) in all nodules, pGGN: 27% (9/36) in all, PSN: 32% (12/36) in all	5.9% (2/34) of all lung cancer patients (65% non-smokers)	50.56
	Liu et al.^128^ (2011) 94-02 screen	China	44% (16/36) non-smoking lung cancer, GGN: 21% (8/38) in all nodules, pGGN: 5% (2/38) in all, PSN: 16% (6/38) in all	5.6% (2/36) of all lung cancer patients (44% non-smokers)	49.43
	Shan et al.^129^ (2020)	Anhui, China	37% (20/54) non-smoking lung cancer, GGN: 27.8% (15/54) in all nodules, pGGN: 16.7% (9/54) in all, PSN: 11.1% (6/54) in all	NA	50.39

GGN, ground-glass nodule; PSN, partial solid nodule; MPLC, multiple primary lung cancers.

Studies summarized here include those in Triphuridet et al.’s meta-analysis with accessible data and Chang et al.’s trial in Taiwan.

Studies without CT feature data for non-smoking lung cancer are ordered by the proportion of non-smokers in their cohort (highest to lowest).

Air pollution data were obtained from the Global Burden of Disease (GBD) database. Expected exposure concentrations were averaged over the 10 years preceding the final date of the study’s screening year. For studies with a screening year earlier than 2000, data from as early as 1990 were used.

2021 WHO Air Quality Guideline Values for PM2.5: the annual guideline value is set at 5 μg/m^3^, while the 24-h guideline value (defined as the 99th percentile, allowing 3–4 exceedance days per year) is 15 μg/m^3^.^130^

Histologically, LUAD is the most frequently reported subtype in PM-associated cases,^88^^,^^112^^,^^132^^,^^133^^,^^134^^,^^135^ with epidemiological studies consistently showing stronger associations between PM2.5/PM10 exposure and adenocarcinomas compared with squamous or small-cell carcinomas.^76^^,^^77^ This trend persists across regions and remains significant among both smokers and non-smokers, although the precise magnitude of risk varies between cohorts.^110^ However, different PM components may influence the carcinogenic effect.^95^ DEPs^91^^,^^95^ and PAHs had been found correlated with squamous and small cell carcinomas.^115^ These PM subtypes or extracts containing high levels of BaP predominantly deposit in the conducting airways, where enzymatic activation by CYP1A1/1B1 generates bulky DNA adducts and induces TP53-associated mutational profiles, promoting proximal bronchial carcinogenesis.^52^^,^^136^^,^^137^^,^^138^^,^^139^ In contrast, PM2.5 enriched in secondary inorganic aerosols (sulfates, nitrates, ammonium) and metals have greater alveolar penetration, which interacts extensively with small bronchiolar and AT2 cells,^12^^,^^140^^,^^141^ thereby favoring adenocarcinoma initiation.^41^^,^^142^ While pathological examination of human samples with definitive PM exposure is still lacking, lung tissue specimens from exposed animals have been repeatedly reported to exhibit several features resembling early premalignant lesions of adenocarcinomas in humans, including progressive alveolar wall thickening, bronchial epithelial hyperplasia,^67^^,^^143^^,^^144^ and expansion of progenitor-like epithelial subsets.^141^^,^^145^^,^^146^

### Molecular features of PM-associated lung cancer

**Figure 4:**
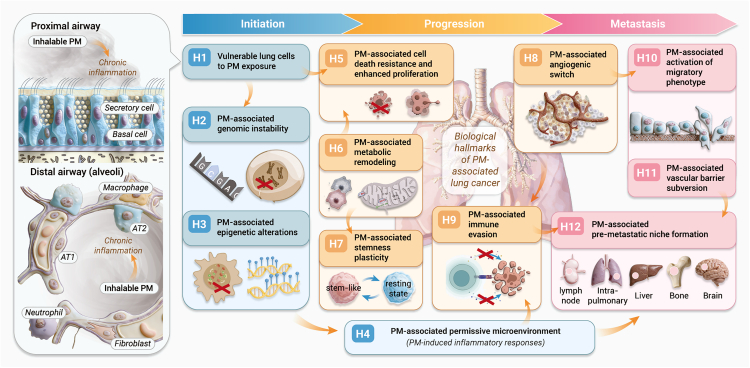
Overview of the integrative model of PM-associated hallmarks across lung cancer evolution This schematic illustrates the 12 hallmarks (H1–H12) presenting a dynamic and interconnected model of lung carcinogenesis organized across three evolutionary stages. This starts with the initiation layer (H1–H4), which establishes a permissive field through cell vulnerability, genomic and epigenetic instability, and a pro-inflammatory microenvironment. The next layer is tumor progression (H5–H9), where survival, stemness, metabolic fitness, angiogenesis, and immune evasion enable robust local expansion, followed by the final layer of metastatic dissemination (H10–H12), orchestrated through epithelial migration potential, vascular barrier subversion, and pre-metastatic niche formation. This integrative view provides a cohesive conceptual lens comprehend the entire evolution of PM-related lung cancer. PM, particulate matter; AT1/2, alveolar type 1 and type 2 cell.

Building upon distinct clinicopathological characteristics, it becomes essential to unravel the biological essences that translate chronic PM exposure into carcinogenesis. Lung cancer arising in PM-exposed individuals emerges with elevated APOBEC-associated mutational signatures, a lack of canonical tobacco-related SBS4,^147^ and more frequent EGFR sensitive mutations.^12^ Rare EGFR variants such as G719X have also been disproportionately detected from patients in areas with heavy indoor coal combustion.^60^ Intriguingly, rather than solely acting as a direct mutagen, *in vivo* evidence proves that PM2.5 exposure can promote carcinogenesis by selectively expanding pre-existing mutant clones through microenvironmental reprogramming.^12^ This points to a complex, hierarchical biological system orchestrated by chronic PM exposure. To synthesize the interconnected pathways into a coherent concept, we propose a framework of twelve hallmarks that delineate the evolution of lung cancer under air pollution (Figure 4, detailed in the following sections), with a three-layer architecture from tumor initiation, progression, to metastasis.

## Hallmarks of PM-associated lung cancer: the initiation phase

**Figure 5:**
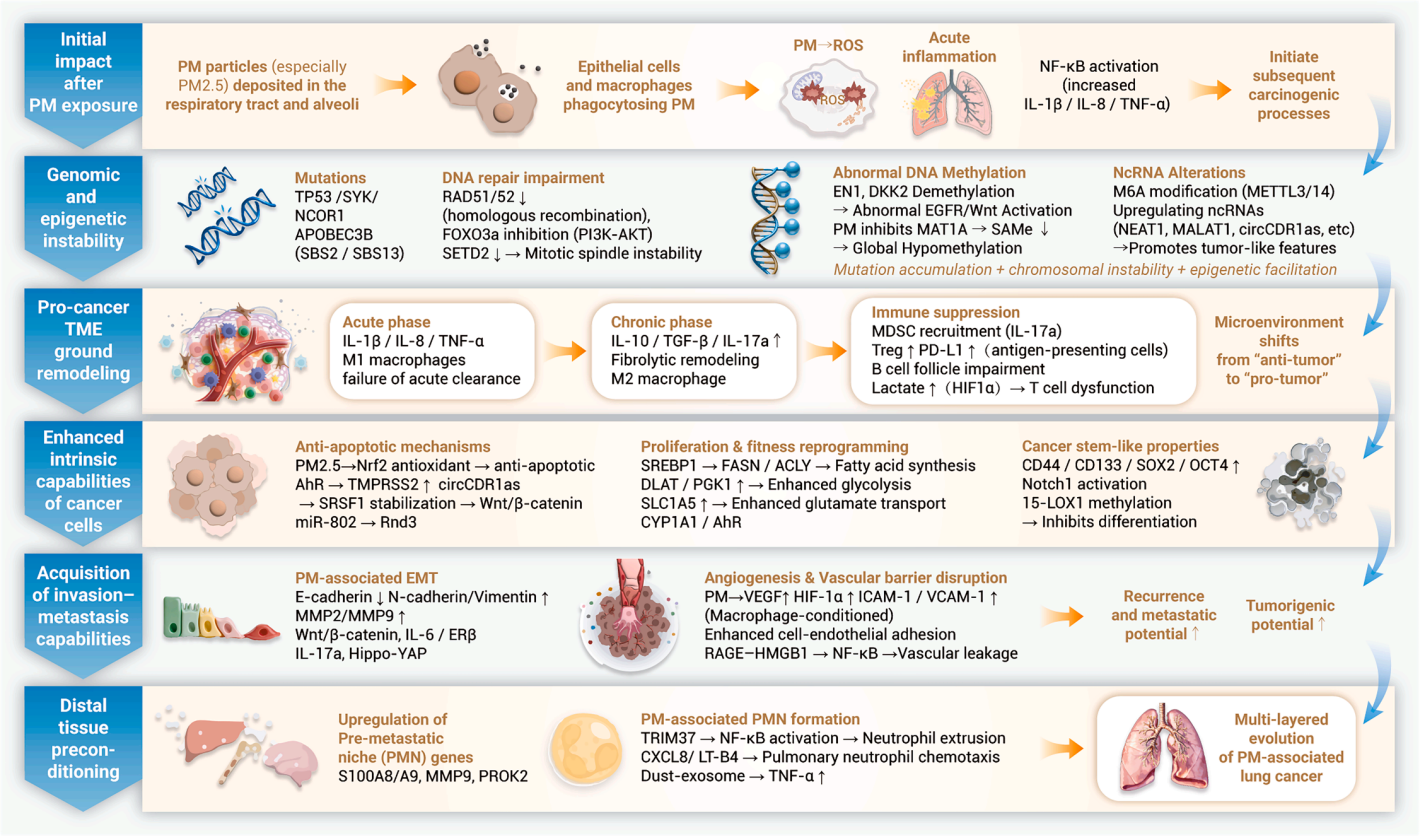
Major pathways involved in the multi-layered evolution of PM-associated lung cancer PM plays a multifaceted role in various stages of lung cancer initiation, progression, and metastasis. PM has the potential to compromise the stability of genetic material within lung epithelial cells, leading to the generation of driver mutations. PM further interacts with a diverse array of cells, fostering tumorigenesis in cells harboring these driver mutations. Concurrently, PM exacerbates the malignant properties of tumor cells and stimulates angiogenesis within the tumor microenvironment. Prolonged exposure to PM also induces immunosuppression. The highly invasive tumor cells breach the vascular barrier influenced by PM, facilitating the establishment of pre-metastatic niches (PMNs) at distant sites, ultimately culminating in the metastasis of the tumor. PM, particulate matter; ROS, reactive oxygen species; Treg. regulatory T cells.

The first four hallmarks establish a permissive environment for tumorigenesis. This phase is initiated by the vulnerability of specific lung cells to PM (H1), which is dramatically exacerbated by the concurrent induction of genomic instability (H2) and epigenetic alterations (H3). These cell-intrinsic changes are synergistically promoted by the permissive microenvironment (H4), driven largely by chronic, non-resolving inflammation.^148^ This milieu not only causes direct tissue damage but also supplies growth signals and suppresses immune surveillance, thereby enabling the survival and expansion of clones harboring PM-associated mutations. Thus, initiation under PM exposure is a concurrent breakdown of multiple cellular safeguards, orchestrated by inflammatory and oxidative signaling (Figure 5; Table 2).^215^^,^^216^

**Table 2:** PM-mediated direct mechanisms of lung cancer

Hallmarks	Subtypes	Mechanisms and experimental model	Combined effect	Note	Ref
Mutagenic or genotoxicity	genetic alteration	*in vitro*: PM induces DNA adducts, double-strand breaks, and oxidative DNA damage, alongside generating APOBEC signatures *in vivo*/population study: The cohort study further validated APOBEC signatures	insufficient evidence	most analyses fail to reveal PM2.5-associated signatures (except those attributed to nitrated PAHs)	Cheung et al.^29^; Borgie et al.^53^; Ndong Ba et al.^54^; Denissenko et al.^137^; Petruzzelli et al.^138^; Yu et al.^139^; Van Den Heuvel et al.^149^; de Oliveira Alves et al.^150^; Xu et al.^151^; Xu et al.^152^; Zhao et al.^153^; Leclercq et al.^154^; Iwai et al.^155^; Abbas et al.^156^; Koh et al.^157^; Chen et al.^158^; Zhang et al.^159^
	epigenetic alteration	*in vitro* and *in vivo*: PM alters DNA methylation and non-coding RNAs in human airway epithelium and lung cancer cells widely			Espín-Pérez et al.^160^; Cayir et al.^161^; Yuan et al.^162^; Clifford et al.^163^; Jiang et al.^164^; Zheng et al.^165^; Li et al.^166^; Zheng et al.^167^
Inflammation	acute exposure	*in vitro* and *in vivo*: PM induces the activation of NF-κB signaling, release of pro-inflammatory cytokines (IL-1*β*, IL-8, IL-12, IL-18, TNF-*α*, IFN-γ) and chemokines, recruitment of neutrophils, and M1 polarization of macrophages	tumor-promoting inflammation	dose, time of exposure, and recovery period affect experimental results	Luo et al.^30^; Chen et al.^65^; Liu et al.^168^; Chao et al.^169^
	subacute or chronic exposure	*in vitro* and *in vivo*: (1) (PM induces) upregulation of anti-inflammatory cytokines (IL-4, IL-10, IL-13, and TGF-*β*); (2) tumor-promoting inflammation including persistent macrophage infiltration and increased IL-1β production; (3) PM-induced systemic responses			Sun et al.^28^; Zhang et al.^170^
Oxidative stressors	oxidative stressors	*in vitro* and *in vivo*: (1) low-dose PM induces low levels of ROS, prompting adaptive antioxidation and enhancing cell viability; (2) high-dose PM induces high levels of ROS, resulting in severe cytotoxicity, while also potentially causing genomic damage			Lovera-Leroux et al.^41^; Leclercq et al.^154^; Abbas et al.^156^; Lin et al.^171^; Liu et al.^172^; Weng et al.^173^; Yun et al.^174^; Xu et al.^175^; Bleck et al.^176^; Natarajan et al.^177^
Immune escape	innate immune	*in vitro* and *in vivo*: (1) PM-exposed macrophages exhibited decreased activation, phagocytic capacity, and altered cytokine production with structural abnormalities in mitochondria and impaired function; (2) macrophages undergo clearance through apoptosis or repolarization toward the inhibitory M2 state; (3) MDSCs are recruited involving the upregulation of IL-17a signaling	suppressive immune microenvironment		Liu et al.^168^; Chang et al.^178^; Ural et al.^179^; Zhang et al.^180^
	adaptive immune	*in vitro* and *in vivo*: (1) PM induces a state of functional exhaustion in the lungs, characterized by elevated Tregs, exhausted T cells, and CD206+ APCs with upregulated PDL1; (2) PM accumulated in human lung lymph nodes significantly disrupts B-cell follicular structures; 3. PM induces an abnormally thickened matrix in lung tissue on the particle surface, hindering the infiltration of cytotoxic CD8^+^ T cells into the lungs			Chang et al.^178^; Ural et al.^179^
Metabolic disorder	lipid metabolism	*in vitro*: PM inhibits 15-LOX1/15-LOX2 and simultaneously activates the *de novo* lipogenesis pathway *in vivo*/population study: BaP-exposed mice reveal disruption of glycerophospholipid metabolism	metabolic disorders toward tumors	the detoxification metabolism of PM itself is involved in promoting lung cancer	Li et al.^136^; Li et al.^166^; Zhu et al.^181^
	carbohydrate metabolism	*in vitro*: PM upregulates ACLY and perturbing energy metabolism (TCA cycle and glycolysis) *in vivo*/population study: PM activates DLAT-mediated glycolysis			Song et al.^39^; Fu et al.^182^; Chen et al.^183^
	amino acid and nucleic acid metabolism	*in vitro* and *in vivo*: PM2.5 perturbs purine metabolism, arginine and proline metabolism, and GSH metabolism			Chen et al.^184^
Tumor cell survival	apoptosis resistance and enhanced proliferation	*in vitro*: (1) Fe^2+^ activates the Nrf2-dependent antioxidation; (2) PAH may activate the AhR leading to the regulation of genes involved in the mitochondrial checkpoint of apoptosis *in vivo*/population study: (1) circular RNA CircCDR1as contributes to PM2.5-induced apoptosis resistance; (2) sunlight-irradiated secondary products of PAHs may further inhibit apoptosis	Enhanced tumor characteristics	Low-dose PM is more potent to induce the CSC and anti-apoptosis phenotype, whereas the acute exposure failed.	Lovera-Leroux et al.^41^; Bayram et al.^63^; Li et al.^141^; Leclercq et al.^154^; Wang et al.^185^; Ferecatu et al.^186^; Teranishi et al.^187^; Xu et al.^188^; Reyes-Zárate et al.^189^; Xu et al.^190^
	CSC properties	*in vitro*: CSC markers and have been detected in PM-exposed lung epithelial cells *in vivo*/population study: xenograft models have demonstrated that PM-induced CSC phenotype enhances the formation, growth, and metastasis of lung tumors			Li et al.^166^; Wei et al.^191^; Jiang et al.^192^; Wang et al.^193^
	angiogenesis promoter	*in vitro*: PM2.5 sustainingly activates the FGF/FGFR/MAPK/VEGF pathway, upregulates HIF-1α and VEGF, and promotes the co-cultured endothelial cells to form vascular-like structures *in vivo*/population study: (1) a dose-dependent relationship between VEGFA expression and PM exposure has been found in lung cancer patients; (2) the above findings in cell models are further validated in subcutaneous xenograft models		PM-induced angiogenesis may require the interaction of microenvironmental cells	Lin et al.^171^; Jiang et al.^192^; Ji et al.^194^; Zheng et al.^195^; Xu et al.^196^
Invasion and metastasis	EMT	*in vitro* and *in vivo*: (1) PM exposure promotes the EMT process of lung cancer cells through non-coding RNA-mediated and other intracellular pathways; (2) PM-induced interactions among lung cells, Th17 cells, and macrophages, facilitating EMT	Invasive features and extensive lung metastases	The regulation of CSCs may be partially independent of the EMT pathway.	Luo et al.^30^; Wang et al.^197^; Yang et al.^198^; Yang et al.^199^; Luo et al.^200^; Deng et al.^201^; Wang et al.^202^; Gao et al.^203^; Liu et al.^204^; Fu et al.^205^; Huang et al.^206^; Park et al.^207^
	intravasation and extravasation	*in vitro* and *in vivo*: (1) PM disrupts the integrity of the endothelial barrier and promotes cancer-endothelial cell adhesion; (2) adhesion molecules (E-selectin, P-selectin, ICAM-1, VCAM-1, PECAM-1) and their receptors are upregulated by PM		insufficient *in vivo* experimental evidence	Zhang et al.^55^; Gao et al.^203^; Olesiejuk and Chałubiński^208^; Soca-Chafre et al.^209^; Gao and Sang^210^; Wang et al.^211^; Lin et al.^212^
	pre-metastatic niches	*in vitro* and *in vivo*: (1) some characteristic genes of PMN formation are significantly upregulated in the lung after PM exposure; (2) PM induces the recruitment of Neutrophils that play a vital role in the formation of lung PMN		The synergy between PM and other components of air pollution has been observed.	Dinh et al.^59^; Li et al.^64^; Liu et al.^172^; Pan et al.^213^; Rocks et al.^214^

PM, particulate matter; APOBEC, apolipoprotein B mRNA editing enzyme; ROS, reactive oxygen species; ACLY, ATP citrate lyase; DLAT, dihydrolipoamide S-acetyltransferase; PAH, polycyclic aromatic hydrocarbons; BaP, benzo[*a*]pyrene; PMN, pre-metastatic niche; CSC, cancer stem cells.

### Hallmark 1: Vulnerable lung cells to PM exposure

The respiratory epithelium comprises diverse cell types, including ciliated epithelial cells, goblet cells, club cells, and alveolar type I (AT1) and type II (AT2) cells, all being potential initiating cells of lung cancer.^148^^,^^217^^,^^218^ Cells harboring driver mutations can exist and expand within healthy tissues without triggering tumorigenesis.^215^^,^^216^ The PM-modified microenvironment as well as cellular reprogramming empower these initiating cells to overcome normal regulatory constraints, thereby driving tumorigenesis. The susceptibility to PM-associated malignant transformation varies by lineages (Table S2).^218^ Among these, AT2 cells have emerged as the key candidate cells-of-origin for LUAD^13^^,^^14^ in the setting of PM exposure.^217^ Studies incorporating conditional, inducible trans-genetic mice and organoids have shown that PM2.5 can induce macrophage infiltration and continuous interleukin-1β (IL-1β) release, reprogramming pre-existing oncogenic AT2 cells into a progenitor-like state capable of initiating tumor development.^12^ In contrast, ciliated or AT1 cells are less susceptible to transformation in similar contexts on male mice.^145^ Microorganisms carried by PM may also play a role in lung tumorigenesis. Adenovirus has been reported to induce the infiltration of leukocytes, primarily T and B cells, contributing to bronchial hyperplasia and adenoma driven by KRAS G12V.^219^

Lineage-tracing in genetically engineered models reinforce the idea that tumor-initiating potential is significantly shaped by both cell identity and the specific driver mutations present. For instance, oncogenic KRAS mutations may generate hyperplastic lesions in club cells or AT1 cells^219^ but may lead to malignant transformation predominantly in AT2 cells.^219^ Similarly, EML4-ALK fusions can induce tumors from AT2 and club cells, but not from the more differentiated ciliated airway or AT1 lineages.^220^ Notably, in PM2.5-exposed *in vivo* models, tumor formation appears contingent on the presence of EGFR L858R mutations,^12^ suggesting that pre-existing oncogenic lesions are necessary but not sufficient for tumorigenesis without a permissive microenvironment. These observations highlight that PM does not function solely as a mutagen but may act as a tumor promoter by altering the behavior of oncogene-bearing progenitor cells, tipping the balance from clonal containment to malignant expansion.

### Hallmark 2: PM-associated genomic instability and mutational signatures

The specific vulnerability of target cells (H1) is further amplified by PM’s ability to cause widespread genomic damage (H2), which arises from oxidative stress, replication stress, and impaired DNA repair.^10^^,^^149^^,^^221^ Studies have demonstrated that PM constituents generate reactive oxygen species (ROS), which induce DNA adducts in human bronchial epithelial cells and blood samples derived from residents in highly polluted areas,^137^^,^^138^ double-strand breaks marked by γ-H2AX,^53^^,^^150^^,^^151^ and oxidative damage, including 8-hydroxy-2′-deoxyguanosine (8-OHdG)^54^^,^^152^^,^^153^^,^^154^ based on *in vitro* and *in vivo* evidence. Such damage can result in may lead to mutations in canonical cancer-associated genes, including TP53, SYK, and NCOR1, particularly with prolonged or repeated exposure.^137^^,^^155^^,^^156^^,^^222^ The genotoxic effects of PM get amplified by its interference with DNA repair machinery and mitotic processes. PM2.5 exposure downregulates homologous recombination mediators RAD51/52^151^^,^^223^ and suppresses forkhead box O3 (FOXO3a) activity via PI3K-AKT signaling^224^^,^^225^ in rats, impairing cellular responses to DNA damage. Synchronously, microtubule stability got disrupted by PM-mediated downregulation of SETD2 in A549, a methyltransferase critical for mitotic spindle formation and chromosomal segregation, further contributing to genomic instability and aneuploidy (Figure 5).^226^

Certain mutational signatures have been associated with PM exposure.^9^^,^^157^ Treatment to PM2.5 extracts for 4 weeks is able to upregulate the APOBEC3B gene in lung epithelial cells and generate APOBEC signatures (32.1% SBS2, 38.1% SBS13, 25.8% DBS2, and 30.0% DBS11),^147^ which have been linked to the onset of lung cancer in non-smokers.^158^^,^^227^^,^^228^ Genomic studies of lung cancer in never-smokers from regions with elevated air pollution reveal a higher frequency of SBS5 clock-like signatures and TP53 mutations.^9^ And the C:G to A:T transversion (85.7%) and XpCpG to XpApG (92.9%) are prevalent signatures among cases with lifetime exposure to coal smoke.^139^ Compared with lung cancer from active smokers, those from non-smokers present more unified mutational patterns^159^ with the absence of tobacco-associated signature SBS4, and significantly lower tumor mutational burden (TMB). While LUAD from smokers show a markedly higher frequency of KRAS mutations and elevated genomic instability, non-smokers are more likely to harbor therapeutically targetable genomic variants (EGFR and ALK) with lower TMB overall.^159^^,^^229^^,^^230^ Additionally, non-smokers’ tumors tend to have shorter telomere lengths, potentially linked to PM2.5-induced telomerase activity.^53^

### Hallmark 3: PM-associated epigenetic alterations

Concurrently with damaging the genome (H2), PM orchestrates a parallel layer of dysregulation that operates above the DNA sequence itself. This takes the form of epigenetic alterations (H3), which also contribute to PM-associated tumorigenesis.^231^ On the one hand, PM dysregulates cancer-associated non-coding RNAs based on population studies^160^ and modulates RNA modifications as N6-methyladenosine (m6A)^161^^,^^162^ in human respiratory epithelial cells, orchestrating post-transcriptional control of oncogenic features. On the other hand, DEP exposure alters DNA methylation patterns,^163^ and benzo(*a*)pyrene of PAHs could lead to hypomethylation of cell proliferation-relevant genes EN1 (Engrailed Homeobox 1) and DKK2 (Dickkopf WNT Signaling Pathway Inhibitor 2)^164^ *in vivo* with validation in Xuanwei residents, and aberrant activation of growth-related pathways such as EGFR signaling (a lung-mimic microfluidic system),^165^ thereby inducing stem-cell-like phenotypes (*in vivo* and *in vitro*).^166^ Oxidative stress-induced depletion of methyl donor availability appears to mediate these epigenetic alterations based on prenatal exposure studies, particularly through inhibition of methionine adenosyltransferase 1A (MAT1A), which reduces levels of S-adenosylmethionine (SAMe),^232^^,^^233^ a universal methyl donor essential for DNA and histone methylation.^234^ Disruption of DNA methyltransferases (DNMTs) and ten-eleven translocation (TET) enzymes has also been documented.^235^^,^^236^ While most evidence comes from preclinical settings, diesel-derived PM exposure has been substantiated to modify DNA methylation in human airway epithelial cells collected via bronchoscopy.^163^ This coordinated disruption of epigenetic homeostasis may create a transcriptional landscape conducive to malignant transformation and clonal evolution.

### Hallmark 4: PM-associated permissive microenvironment

The cell-intrinsic damage caused by PM-associated disruptions to genome and epigenome (H2-H3) does not occur in isolation, it is embedded within and powerfully reinforced by a permissive microenvironment (H4). While investigators in the last decade largely attributed PM’s carcinogenic potential to its mutagenic effects, accumulating evidence reveals a broader spectrum of pro-tumorigenic activities. The inflammatory response elicited by PM exposure emerges to play a central role in shaping a tumor-permissive lung microenvironment.^237^ Figure 2 and Table S3 summarize the reported biological effects of PM on the lung. Inhaled PM activates pattern recognition receptors and pro-inflammatory cascades, resulting in acute the activation of NF-κB signaling and the delivery of pro-inflammatory cytokines, including IL-1β, IL-8, IL-12, IL-18, TNF-α, and IFN-γ, recruitment of neutrophils, and M1 polarization of macrophages.^65^^,^^168^ According to *in vivo* observations, the internal processes initiated by PM2.5 starts with airway clearance and enzymatic degradation,^238^ which usually fail to eliminate PM. Subsequently, persistent PM deposition in the lung leads to long-term retention within alveolar macrophages and extracellular matrices, sustaining chronic inflammation even after cessation of exposure.^12^^,^^239^ While acute inflammation may initially suppress tumorigenesis via cytotoxic responses, chronic low-dose or subacute exposure to PM gradually shifts the microenvironment toward an immunoregulatory, pro-tumorigenic state.^170^^,^^240^ This chronic phase is characterized by increased levels of anti-inflammatory cytokines, such as IL-10, IL-4, and TGF-β, along with the expansion of regulatory immune cell subsets, and remodeling of tissue architecture.^65^^,^^168^^,^^241^ Inflammatory mediators IL-1β have been shown to directly reprogram AT2 cells with latent driver mutations, facilitating their transition into tumor-initiating phenotypes.^12^ Cytokines IL-6^30^ and IL-17a^169^ stimulated by PM2.5, which possess both pro- and anti-inflammatory effects, have been found to directly enhance cell proliferation and xenograft tumor growth (Figure 5). Apart from local inflammation of the respiratory tract, PM-induced chronic systemic responses participate in lung carcinogenesis. Plasma from individuals with occupational PM exposure can activate oncogenic signaling pathways in bronchial epithelial cells, implicating circulating factors in promoting distal carcinogenesis.^170^

Emerging evidence has revealed a dynamic landscape of PM-induced inflammatory responses, yet unfortunately, only a few studies have introduced recovery periods (Figure 2). Therefore, we continue to categorize exposure durations to facilitate the comparison among experiments, delineating acute, subacute, and chronic exposure by setting boundaries at 1 and 4 weeks in protocols. Figure 2 highlights situations closely resembling real-world exposure, including low-dose chronic exposure, ambient chronic exposure, and high-dose or acute exposure followed by a prolonged recovery period. In general, the biological effects of PM observed in previous experiments exhibit heterogeneity. Nevertheless, when crudely compared with other scenarios, real-world-like exposures recapitulate tumor-promoting phenotypes more consistently than acute high-dose models, reinforcing the relevance of cumulative exposure and inflammatory memory in lung cancer initiation.

The dual role of ROS in inflammation and DNA damage further amplifies the oncogenic potential of PM. Dose-dependent effects have been observed. Moderate ROS levels induced by subtoxic PM exposure can stimulate proliferation and adaptive antioxidant responses in pre-malignant cells.^41^^,^^154^^,^^242^ Conversely, higher levels of ROS induced by elevated levels of exposure result in severe cytotoxicity but may induce tumorigenesis by causing genomic damage.^38^^,^^155^ PM-induced generation of ROS also exacerbates the inflammatory responses by chemoattracting neutrophils and monocyte-derived macrophages,^171^^,^^172^ upregulating the NF-κB pathways,^173^^,^^174^ releasing inflammatory factors^175^^,^^176^^,^^177^ both *in vitro* and *in vivo*. The interplay between inflammation, oxidative stress, and epithelial cell reprogramming thus constitutes an essential axis in PM-mediated lung tumor initiation.

## Hallmarks of PM-associated lung cancer: the progression phase

The subsequent five hallmarks propel the evolution of established lesions into clinically significant tumors by conferring key cancer capabilities. This phase is driven by a self-reinforcing cycle of cell survival and proliferative signaling (H5), which is fueled by profound metabolic remodeling (H6) that provides the necessary bioenergetic and biosynthetic substrates. Concurrently, tumors under PM pressure enhance their regenerative potential by acquiring stemness plasticity (H7). To support this expanding tumor mass, PM exposure triggers an angiogenic switch (H8), ensuring a dedicated blood supply. Critically, these processes unfold within an immune evasion circuitry (H9), where PM-induced immunosuppression creates a permissive niche for local expansion and invasion. Thus, progression under PM exposure represents the co-opting of fundamental physiological processes, metabolism, stemness, and angiogenesis, within an immunologically shielded environment, enabling robust tumor growth (Figure 5; Table 2).^243^

### Hallmark 5: PM-associated cell death resistance and enhanced proliferation

**Figure 6:**
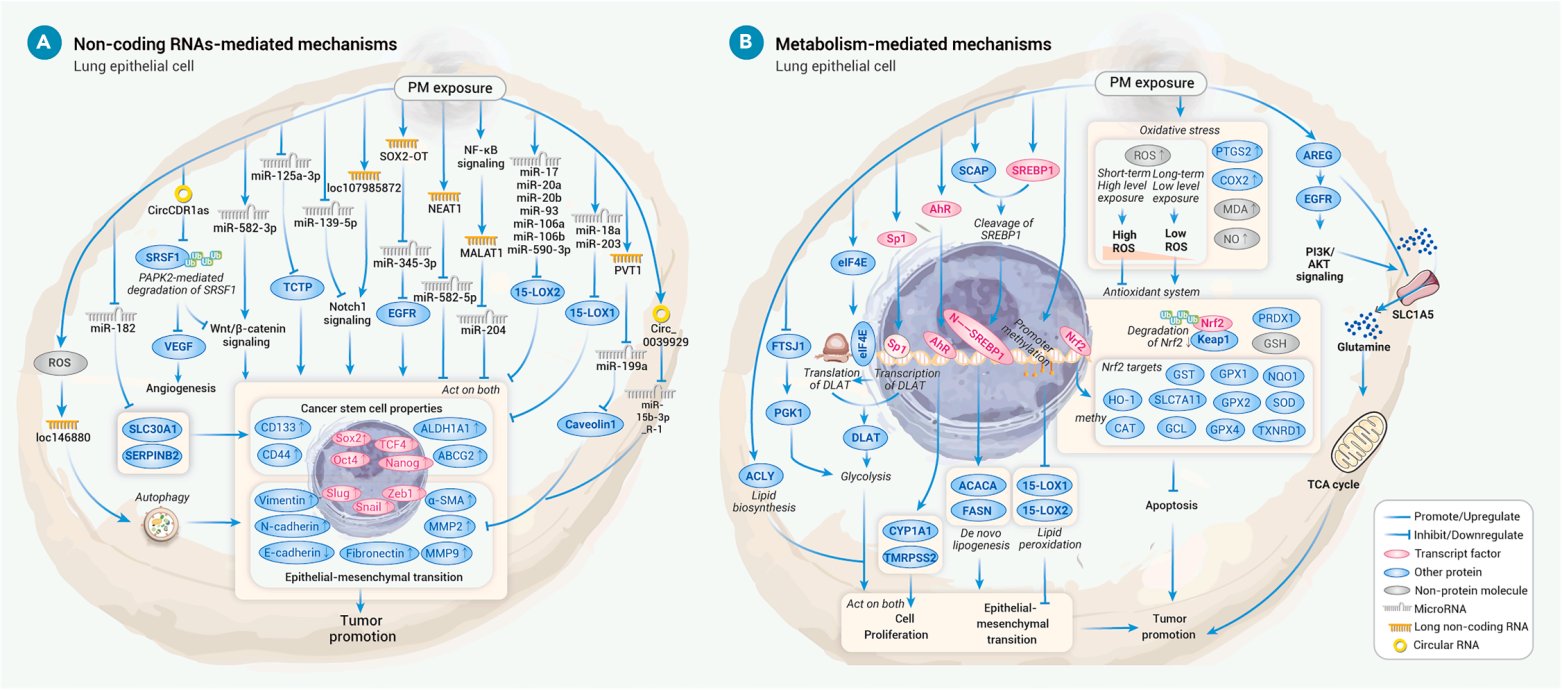
Epigenetic and metabolic mechanisms underlying the progression of PM-associated lung cancer (A) PM upregulates the expression of genes and transcription factors associated with tumor stem cells and epithelial-mesenchymal transition via multiple non-coding RNAs, thereby enhancing tumor invasiveness. (B) PM influences glucose, glutamine and lipid metabolism, augmenting tumor proliferation and epithelial-mesenchymal transition. Furthermore, chronic exposure to PM stimulates the antioxidant system via sustained low-dose ROS, ultimately suppressing tumor cell apoptosis.

PM exposure promotes tumor progression by tipping the balance toward survival and proliferation. Although PM induces apoptosis in most experiments, it can suppress the death of normal and cancerous respiratory epithelial cells under certain circumstances depending on the composition and exposure schemes. Components have been identified to elucidate the anti-apoptotic effect of low-dose PM2.5 through parallel mechanisms. Fe^2+^ activates the NRF2 (nuclear factor erythroid 2-related factor)-dependent antioxidation in human bronchial epithelial cells,^41^^,^^154^ while PAHs upregulate TMPRSS2 (transmembrane serine protease 2) by inducing the translocation of AhR (aryl hydrocarbon receptor) in several lung cancer cell lines and exposed mice.^185^^,^^186^ Additionally, sunlight-irradiated secondary products of PAHs may further inhibit apoptosis by producing ROS.^187^ Non-coding RNAs are also implicated in PM-associated apoptosis resistance through typical “sponge” or atypical roles (Figure 6). For instance, PM2.5 suppresses miR-802 to upregulate Rnd3, promoting tumor growth in a xenograft model with long-term exposure but enhancing cell death as well as lung injuries within 7 days in mice.^145^ Meanwhile, the circular RNA CircCDR1as contributes to PM2.5-promoted apoptosis resistance and tumor growth by binding to SRSF1 (serine/arginine-rich splicing factor 1) and strengthening wnt/β-catenin signaling.^188^ The magnitude of these effects appears dose dependent (Figure 2). Intermediate PM concentrations elicit the most pronounced anti-apoptotic responses, while excessive exposure may trigger necrosis or growth arrest. The anti-apoptotic ability of diesel-derived PM is strongest at 10 μg/mL compared with 5 or over 50 μg/mL.^63^ And exposure to 10 μg/cm^2^ of PM10 fosters apoptosis evasion in lung cells by STAT3 activation.^189^ These findings indicate the significance of low-dose exposure in mediating the anti-apoptotic effects of PM.

Another consistently observed feature in PM-exposed experiments is enhanced proliferation. Single-cell analysis of mice exposed to PM2.5 revealed that all eight subtypes of lung epithelial cells exhibited increased proliferation after 1 month of exposure, with AT2 and goblet cells showing particularly heightened proliferation rates^141^ PM promotes cell proliferation *in vitro* and accelerates tumor growth *in vivo* through the modulation of various signaling cascades. Apart from pathways mentioned above, these also include the activation of miR-200a-3p-TNS3 axis^244^ and hnRNPA2B11-dependent signaling,^245^ while suppressing negative regulators such as Engrailed1-Wnt signaling^164^ and the lncRNA SPRY4-IT1-DUSP6-ERK1/2-Chk1 pathway.^246^ Inflammatory mediators from immune cells further amplify these effects. PM2.5 exposure stimulates IL-17a secretion by Th17 cells^169^ and IL-1β release from macrophages,^12^ both of which play a role in promoting lung cancer progression *in vivo*. Besides, the tumor growth promoting effects of Wnt3a-enriched exosomes from PM-treated cells suggests a self-reinforcing mechanism that sustains proliferation through paracrine signaling.^190^

### Hallmark 6: PM-associated metabolic fitness remodeling

Together with heightened demand conferred by proliferative advantage (H5), tumor cells undergo metabolic fitness remodeling relevant to PM exposure (H6), fundamentally rewiring their nutrient acquisition and utilization pathways to fuel their expansions (Figure 6).^247^ Lipid metabolism is a major target of PM-associated reprogramming.^248^ PM2.5 activates the cleavage and nuclear translocation of SREBP1, upregulates ATP citrate lyase (ACLY), and increases the upregulation of fatty acid synthase (FASN) and acetyl-CoA carboxylase, collectively enhancing lipogenesis *in vivo* and *in vitro*, which further fuels tumor progression.^181^^,^^182^ These changes are coupled with reduced peroxidation of polyunsaturated fatty acids in the respiratory epithelium, mediated by the downregulation of 15-lipoxygenases through CpG methylation and miRNA modulation, thereby stabilizing membrane integrity and supporting cell growth.^166^ Glycerophospholipid could influence lipid distribution and metabolism through their association with lipid transfer proteins.^249^ Untargeted lipidomic analysis of serum from benzo[*a*]pyrene-exposed mice reveals disruption of glycerophospholipid metabolism, akin to those observed in lung cancer.^136^ Interestingly, a gender difference was noted, with phosphatidylinositol metabolism showing greater susceptibility to exposure in males.^136^

PM also impacts glucose metabolism and the Warburg effect. PM2.5 enhances glycolysis in lung cancer cells through upregulation of DLAT (dihydrolipoamide S-acetyltransferase), driven by transcriptional and translational regulation via Sp1 and eIF4E. This leads to increased proliferation, migration, and resistance to apoptosis. Clinically, elevated DLAT expression correlates with poor prognosis and increased radiolabeled glucose uptake on PET/CT scans.^183^ PM2.5 also upregulates the ATP citrate lyase (ACLY), a critical regulator linking the high rates of glycolysis and *de novo* lipid synthesis in cancer cells, with xenograft verification of promoting tumor progression.^182^ Moreover, PM2.5 upregulates phosphoglycerate kinase 1 (PGK1) by suppressing the tRNA methyltransferase FTSJ1, further promoting glycolytic flux and malignancy in NSCLC.^250^ Notably, compositions of PM2.5 modulates these observed effects, with organic components upregulating glycolysis in BEAS-2B cells while water extracts may have the opposite effect.^39^

Recent studies indicate that amino acid metabolism may also be affected by PM exposure. In A549 cells, PM enhances AREG expression by activating the EGFR-PI3K-AKT-mTOR pathway, which in turn induces the expression of the glutamine transporter SLC1A5. This facilitates mitochondrial respiration through intermediates derived from the glutamine-driven TCA cycle, ultimately driving cell proliferation *in vitro* and *in vivo*.^184^^,^^251^ The detoxification metabolism of PM can also promote lung cancer. AhR and cytochrome P450 1A1 (CYP1A1) induced by PM2.5 confer higher susceptibility to transform lung epithelial cells.^184^

### Hallmark 7: PM-associated stemness plasticity

PM promotes a cancer stem cell (CSC)-like phenotype, which contributes to tumor propagation, metastasis, and resistance to therapy.^252^ Markers associated with stemness (e.g., CD44,^191^ CD133,^192^ ABCG2,^191^ SOX2,^213^ and OCT4^193^) are upregulated in PM2.5-exposed lung epithelial cells and animal lungs. Functional assays demonstrate increased tumor-sphere formation and enhanced tumorigenicity in xenograft models.^213^ Mechanistically, PM exposure suppresses differentiation-promoting pathways and favors epigenetic and transcriptional programs associated with self-renewal. For example, water extracts of PM2.5 have been shown to promote CSC characteristics by suppressing 15-lipoxygenase expression through methylation.^166^ Other implicated pathways include the activation of the Notch1 signaling pathway^193^ and the NEAT1 (Nuclear paraspeckle assembly transcript 1, one lncRNA)-miR-582-5p axis.^192^ Additionally, low-dose PM2.5 exposure appears more effective in inducing CSC traits in A549 cells than acute high-dose exposure,^191^ highlighting the relevance of sustained subclinical environmental pressure in shaping tumor plasticity.

### Hallmark 8: PM-associated angiogenic switch

As the tumor bulk expands, driven by proliferative, metabolic, and stem-like capabilities (H5-H7), angiogenic switch is gradually triggered, where PM exposure stimulates the formation of new vasculature to deliver oxygen and nutrients (H8), thereby averting starvation and supporting continued growth.^194^ A dose-dependent positive correlation between VEGFA expression and PM2.5 exposure has been found in lung cancer patients.^194^ In preclinical studies, PM2.5 substantially activates the FGF/FGFR/MAPK/VEGF pathway of lung epithelial cells,^195^ upregulates HIF-1α and VEGF (vascular endothelial growth factor),^171^^,^^192^^,^^196^^,^^253^ and promotes the co-cultured endothelial cells to form vascular-like structures.^194^ These findings are further validated in subcutaneous xenograft models of A549 cells exposed to PM.^194^ In addition, PM-associated angiogenesis may require interaction with microenvironmental cells. The elevation of angiogenic factors in lung cancer cells and increased micro-vessel density in xenografts were detected only when supernatant from PM2.5-exposed macrophages was added.^254^ This suggests that PM-associated angiogenesis is not only driven by tumor cell-intrinsic programs but also relies on crosstalk with immune and stromal cells.

### Hallmark 9: PM-associated immune evasion circuitry

Beyond affecting the intrinsic aggressiveness of cancer cells (H5-H7), PM exposure also plays a role in shaping the extrinsic microenvironment into immunosuppressive (H9) by modulating the physiological and physicochemical properties of immune cells.^168^ Chronic PM2.5 inhalation disrupts both innate and adaptive immune compartments.^168^ Alveolar macrophages gradually lose phagocytic function and exhibit mitochondrial damage when chronically loaded with indigestible PM.^178^^,^^179^ Long-term PM2.5 exposure also recruits myeloid-derived suppressor cells (MDSCs) through IL-17a signaling, further enhancing immune suppression.^180^ For adaptive immunity, exposure to the carbon black components of PM elevates the proportion of both regulatory T cells (Tregs) and exhausted T cells *in vivo*, and bulk PM2.5 exposure enhances the expression of immune checkpoint proteins such as PD-L1^12^ on antigen-presenting cells.^255^^,^^256^ In human lung lymph nodes, disrupted B cell follicular structures have been reported to be associated with inhalable PM deposition.^179^ Physicochemical effects of PM further exacerbate immune evasion. PM1 can adsorb peroxidasin on its surface, contributing to the formation of abnormally dense extracellular matrices that physically restrict cytotoxic CD8^+^ T cell infiltration.^257^ Furthermore, lactate production resulting from PM-activated HIF1α pathway in lung macrophages acidifies the local tumor microenvironment (TME), creating metabolic conditions unfavorable for effector T cell function.^178^ These findings underscore that PM-induced immune dysfunction is not limited to individual cell populations but represents a coordinated suppression of immune surveillance at multiple levels.

## Hallmarks of PM-associated lung cancer: The metastasis phase

The final three hallmarks orchestrate the dissemination of lung cancer, representing the most lethal dimension of PM-associated carcinogenesis. This phase is initiated by the activation of a migratory phenotype (H10), primarily through epithelial-mesenchymal transition, which enables tumor cells to detach from the primary mass and invade surrounding tissues. To enter the circulation, these motile cells exploit PM-induced vascular barrier subversion (H11), wherein endothelial dysfunction enhances permeability and facilitates intravasation. Ultimately, PM’s effect in fostering pre-metastatic niche formation (H12) allows successful colonization of distant organs, creating a receptive microenvironment in advance of circulating tumor cells.^258^ These processes establish a biological continuum from local progression to systemic spread (Figure 5; Table 2).

### Hallmark 10: PM-associated activation of migratory phenotype

The EMT represents a vital mechanism through which cancer cells shed epithelial traits and develop migratory properties, which are essential for metastasis. Exposure to PM has been demonstrated to promote EMT in lung cancer via both intracellular and intercellular mechanisms (Figure 5). At the molecular level, EMT pathways such as Notch1,^197^ Smad1 signaling,^198^ Wnt/β-catenin,^199^ Hippo-YAP, miR-204/ZEB1, and PI3K/Akt^30^^,^^200^^,^^201^^,^^202^^,^^203^ are activated by PM exposure in lung cell lines (Figure S4). PM also remodels the competing endogenous RNA (ceRNA) networks to regulate EMT-related transcription factors and signaling cascades. Canonical ceRNA axes discovered *in vitro* include miR-125a-3p/TCTP (translationally controlled tumor protein),^204^ PVT1 (plasmacytoma variant translocation 1, one lncRNA)/miR-199a/caveolin1,^259^ SOX2-OT (SOX2 overlapping transcript, one lncRNA)/miR-345-5p/EGFR,^205^ MALAT1 (metastasis-associated lung adenocarcinoma transcript 1, one lncRNA)/miR-204,^200^^,^^260^ and circ_0039929/miR-15b-3p_R-1.^206^

Beyond cell-intrinsic regulation, PM also promotes EMT through microenvironmental cues involving homologous or heterogeneous cellular interactions. For example, PM2.5 stimulates IL-6 secretion via estrogen receptor β (ERβ) activation, which can act via autocrine or paracrine loops to reinforce mesenchymal transitions.^30^ IL-17a derived from Th17 cells^169^ and heparin-binding EGF-like growth factor (HBEGF) released by macrophages activated by PM further enhance this process, promoting tumor metastasis^207^ *in vivo*. These findings suggest that PM fosters EMT not only through direct transcriptional control but also by orchestrating a supportive inflammatory milieu^261^

### Hallmark 11: PM-associated vascular barrier subversion

After acquiring an invasive phenotype (H10), PM contributes by increasing endothelial permeability and disrupting vascular integrity (H11), which facilitates tumor cell entry into the circulation and survival within the bloodstream.^208^ Simultaneously, PM upregulates adhesion molecules on endothelial surfaces and their corresponding ligands on tumor cells,^55^^,^^209^ enhancing both vascular attachment and transendothelial migration. These changes confer survival advantages to circulating tumor cells (CTCs).^262^ PM-induced expression of platelet-binding receptors in lung cancer cells may stabilize CTCs within the bloodstream, shielding them from immune recognition and shear stress,^209^ which still requires validation *in vivo*. Furthermore, PM2.5 augments interactions between tumor cells and endothelial cells in coculture models^203^^,^^210^ by elevating early adhesion molecules (E-selectin, P-selectin) and their receptors, as well as late adhesion molecules (ICAM-1, VCAM-1, PECAM-1)^55^^,^^150^^,^^203^^,^^211^^,^^212^ and receptors,^55^^,^^209^ promoting extravasation and subsequent metastatic colonization. Additionally, PM-induced inflammation participates in promoting adhesion. The uptake of quasi-superfine particles triggered the release of HMGB1 (high-mobility group box 1), activated NF-κB-mediated production of pro-inflammatory cytokines via interaction with RAGE (receptor for advanced glycation end-products), leading to enhanced cancer-endothelial cell adhesion.^210^ Another *in vivo* experiment of PM2.5 confirmed the role of autophagy-mediated upregulation of ICAM-1.^211^

### Hallmark 12: PM-associated pre-metastatic niche

While successful intravasation (H11) places tumor cells into systemic circulation, their journey is futile without a receptive destination. Remarkably, PM exposure systemically pre-programs distant sites by fostering the formation of a pre-metastatic niche (PMN) (H12), creating a hospitable soil that supports the seeding.^243^ PMN-associated genes (PROK2, S100A8, S100A9, and MMP9) get upregulated in lung tissue within days of PM exposure, suggesting early remodeling of distal sites.^171^ PM-driven neutrophil recruitment into pre-metastatic tissues is central to PMN formation. Besides the canonical CXCL8 (IL-8)-mediated chemotaxis, some non-canonical mechanisms have been stimulated. In lung epithelial cells, PM triggers the autophagic degradation of TRIM37 (tripartite motif-containing 37), stabilizing TRAF6 (TNF receptor-associated factor 6), subsequently activating NF-κB-driven neutrophil infiltration and vascular leakage.^172^ Diesel-derived PM activates leukotriene B4 signaling to enhance neutrophil infiltration in mice lung,^64^ while ozone synergizes with PM by promoting neutrophil extracellular trap (NET) release.^214^ Meanwhile, extracellular vesicles isolated from indoor dust promote lung metastasis by inducing TNF-α, possibly involving the migration of neutrophils.^59^

Although PM-associated PMN mechanisms in pulmonary metastasis are increasingly defined, their extrapulmonary relevance remains poorly understood. Limited evidence suggests that PM2.5 may enhance the CSC traits to facilitate liver or kidney metastasis in patient-derived xenograft models,^213^ yet the organotropism and molecular determinants of PM-associated PMN remain elusive. Given the distinct patterns of intrapulmonary and extrapulmonary metastasis^263^^,^^264^ observed in non-smoking lung cancer,^265^ further investigation is warranted to delineate how PM exposure influences metastatic trajectories across different anatomical sites.

## An integrative hallmark framework for PM-associated lung cancer

Taken together, the 12 hallmarks of PM-associated lung cancer illustrate a progressive transition from reversible cellular stress responses to stable malignant programs. At the primary layer of initiation, PM’s physicochemical features directly provoke epithelial injury (H1), DNA and epigenetic damage (H2, H3), and form a permissive microenvironment (H4), thereby establishing the initial vulnerability of lung epithelium that facilitates aberrant clonal expansion. These early perturbations activate the second layer of progression, characterized by selection of aggressive cell states (H5, H7), metabolic and mitochondrial stress (H6), stromal and angiogenic reprogramming (H8), and altered immune responses (H9). These changes cooperate to form the thrust of tumor progress. Ultimately, the downstream layer of metastasis encompasses migratory programs (H10), vascular barrier disruption (H11), and pre-metastatic niche formation (H12). Notably, these layers are not completely linear steps but mutually reinforcing circuits, with chronic inflammation as a major connector dynamically interacting with most hallmarks. For example, PM-induced epithelial injury elicits macrophage-dominant inflammation, which further accelerates epithelial plasticity.^12^ These cross-regulatory loops create a self-perpetuating carcinogenic ecology.

The hallmark framework also provides a coherent biological basis for the nonlinear exposure-response patterns observed in epidemiological studies. At low to moderate PM2.5 concentrations (<30 μg/m^3^), PM dose proportionally contributes to genomic instability (H2), epigenetic alterations (H3), and a pro-inflammatory microenvironment (H4), allowing incremental increases in exposure to produce biological injury conforming to a near-linear relationship. At moderate concentrations (30–60 μg/m^3^), the synergistic engagement of progression hallmarks (H5-H9) gives rise to the transition to a supra-linear acceleration, where positive feedback loops form by enhancing survival pathways (H5) and stemness (H7), driving metabolic fitness (H6), and enforcing immune evasion (H9), thereby amplifying tumor promotion beyond simple additive effects. Finally, the saturation of risk at very high exposure levels (>60–75 μg/m^3^) likely results from the biological exhaustion of these systems, including maximal immune suppression (H9), and depletion of susceptible cellular progenitors by dominant toxicity, collectively leading to a plateau in population-level risk.

## Towards better insights into PM mechanisms on carcinogenicity

The hallmarks synthesize a vast body of mechanistic evidence. To move from descriptive observations to mechanistic advances, here we propose a research agenda built around unresolved questions, testable hypotheses, and conceptual guidance for study design and data integration.

### Unsolved questions in PM carcinogenicity

Several issues need to be addressed. Foremost is the specific cellular origins and immune cell collaborator for PM-associated lung carcinogenesis. While research has revealed EGFR-mutant AT2 cells for LUAD origin and alveolar macrophages as mediators,^12^ PM-associated tumorigenesis may involve a broader spectrum of lung cell types with varying mutations and immune cell companions (see the next section). Conditional and inducible genetically engineered models that control the expression of driver mutations in specific cell types, as well as lineage-tracing technologies, coupled with depletion or adoptive transfer of specific immune cells, are required for ongoing experiments. Another ambiguity is the time dynamics and reversibility of PM-associated molecular alterations. Continuous sampling throughout the time course of PM intervention covering peri-exposure periods (>1–3 months before and after exposure) is required to quantify the latency between PM, molecular remodeling, and clonal expansion, along with the reversible extent of these changes after exposure reduction. It is also broadly unknown how the influence of PM on the outside of the lung and the whole body promote metastasis, which could be characterized by liquid biopsies and tissue sampling from distal organs^266^ Additionally, while the synergistic effects between smoking and PM exposure have been observed in epidemiological studies, the underlying mechanisms and cancer patterns among smokers exposed to different levels of PM remain unexplored, requiring comparative validation between the co-exposure model and the single-exposure model. Furthermore, indoor PM is underrepresented in lung cancer biology. Cooking emissions can reach extremely high concentration in a short time and contain reactive organic compounds.^29^^,^^56^^,^^267^ However, most mechanistic investigations lack direct comparison between indoor and outdoor PM, and few population-level exposure assessments separately quantify indoor PM concentrations.^69^^,^^268^ Dedicated studies using personal exposure monitoring and real-time indoor air sensors can aid in addressing these concerns.

### Experimental designs to explore PM mechanisms

#### Optimizing the laboratory modeling of PM exposure

Experimental models to understand the carcinogenic role of PM basically involve the dosages, exposure frequency and duration of PM exposure as well as the exposed object. Based on the equivalent atmospheric inhalation concentrations (μg/m3) for PM calculated from 75 animal-based studies,^269^ intra-respiratory tract instillation leads to significantly higher exposed doses compared with levels reported in polluted areas by WHO, possibly inducing pronounced cell death upon PM exposure. This standardized comparison then concluded that inhalation of PM in environmental air concentrations is the preferred protocol, and alternatively an appropriate dose for instillation experiments is less than 170 μg/kg bw, only around one-third of what has been traditionally used.^269^ As for time course, most of the published designs adopted acute periods or immediate endpoint assessment on cells taken during PM exposure or animals sacrificed right after exposure. This may lead to disparities from the responses observed under real-world exposure, where post-exposure repair works.^62^^,^^67^ Given PM’s cumulative biological impact persisting at least 2^67^ to 7^12^ weeks since exposure cessation, future investigations are recommended to integrate extended recovery periods into experimental timelines. Interactions between dose and duration of PM also require address to capture the combined effects.

While previous studies on PM2.5 usually used 2D cell culture models,^270^ this approach does not accurately replicate actual lung exposure.^271^ Instead, three-dimensional cell culture systems and organoids offer more physiologically relevant representation of cellular responses.^260^^,^^272^^,^^273^ When using xenograft models, it is recommended to carry out two experimental schemes in parallel, transplantation of exposed cancer cells into mice to assist validating the PM-associated alterations in cell behaviors, and adding ambient exposure of mice to illustrate malignant transformation under real-world-like exposure.

#### Overcoming experimental challenges derived from PM itself

As discussed before, the physiochemical properties of PM extensively affect the carcinogenic effect. The composition of PM used varies across studies in particle size,^34^ weather^274^ (seasons),^55^ chemical properties, solvents, and sources.^275^ Researchers should clarify these factors when describing experiments and comparing their conclusions with others (Table 3). While purification of specific components risk overlooking the synergistic interactions, it is recommended conduct in parallel to the experiments of bulk PM mixture, along with correlation analyses between major components and biological effects.^29^ Recent advances in ALI models further enabled simultaneous chemical analysis of PM composition and biological signal detection.^195^ Given the confounding variation of primary pollutants across countries, regions,^15^ and genders,^38^ multi-factor adjustments are necessary to improve comparability. Meanwhile, immune responses arising from different sizes of PM remain to be elucidated.

**Table 3:** Recommendations for experimental design of PM mechanisms

Factors affecting outcomes		Corresponding recommendations
Model objects	cell lines	(1) use lung cell lines derived from: lung cancer of different pathology and with different driver mutations, normal alveolar and bronchial epithelium
		(2) include non-cancerous cells: co-culture, supernatant addition
		(3) use lung cells and immune cells isolated from animal models and individuals if possible
		(4) knockout or knockin experiments when validating the potential target molecules (instead of inhibitors)
	animals	(1) inducible and conditional transgenic models are preferred to realize the tissue-specific temporal control of gene expression (e.g., Cre/*LoxP* system)
		(2) xenograft models: PDX preferred, use CDX from subcutaneous and intravenous injection
		(3) include rats and mice, of both sexes if possible
		(4) include blood tests and sampling from extrathoracic organs
		(5) incorporate lineage-tracing technologies to track cell fate and contribution of specific cell populations
	individuals	(1) include lung cancer patients with different pollution levels: tumor samples, tissues adjacent to cancer, and distant normal tissues
		(2) include healthy individuals with different pollution levels
		(3) see Figure 7 for advice on clinical investigations
		(4) conduct long-term PM monitoring in the environments of individuals
Exposure regimens	compositions	(1) parallel design: total PM, water extracts, organic extracts
		(2) clarify the chemical compositions of collected PM, or use PM that is commercialized or whose composition has been reported
		(3) report the details of PM collection: source, collection site, season, day or night, traffic conditions, industrialization degree, particle size, and preparation process
	dose	pre-experiment to determine proper doses to maintain cell viability
	mode	(1) *in vitro*: microfluidic system, organoids
		(2) *in vivo*: real-world-like ambient exposure with dynamic recording of PM levels is preferred if accessible
		(3) concentrated PM inhalation or intratracheal instillation: control the liquid amount and PM concentration (better to calculate based on the published formulas)
	duration	(1) long-term exposure (≥1 month) and short-term exposure followed by recovery periods
		(2) capture the interaction of duration and dose (e.g., exposure time-dose curve)
Others		(1) use published datasets: potential target mining, result validation
		(2) include high-throughput multi-omics (bulk-sequencing, single-cell sequencing, spatial-omics) and bioinformatic analyses
		(3) rigorous construction of evidence chain: can each “node” on the chain lead to the final phenomena?

CDX, cell line-derived xenograft; PDX, patient-derived xenograft.

PM inherent properties also generate alarms in data analysis and interpretations. For example, PM cytotoxicity may cause bias in cell isolation and downstream single-cell analysis, and the color of PM could distort fluorescent-based experiments. To minimize these impacts, Tissue-resolved molecular techniques such as mass spectrometry imaging (MSI) or spatial transcriptomics provide more reliable analyses while accounting for PM physiochemical properties.^276^^,^^277^ Unlike bulk assays, MSI preserves the spatial location of inhaled particles and can detect metal ions, lipid peroxidation products, or adsorbed organic compounds directly within lung tissue, thereby linking specific molecular species carried by PM to localized epithelial injury, metabolic stress, or inflammatory activation.^278^ This approach avoids the confounding effects when comparing samples with varying particle sizes or chemical compositions. Spatial transcriptomics further resolves PM-exposed cellular niches without dissociating tissue architecture, minimizing loss of PM-cell contact information.^279^ This technique helps capture cell-type-specific transcriptional programs within defined PM-deposited microdomains and allows quantification of distance-dependent cellular responses such as gradients of AT2 cell plasticity, which cannot be resolved through homogenized tissue profiling.^279^ Together, coupling MSI and spatial omics provides a chemically informed, spatially resolved reconstruction of how PM interacts with lung microenvironment to promote carcinogenesis.

### Proposed mechanistic hypotheses to address immune gaps in PM carcinogenesis research

As mentioned above, existing PM literature disproportionately emphasizes tumor-intrinsic carcinogenic mechanisms (H1-H3, H5-H8, H10-H11), largely because these pathways are more experimentally accessible while the essential role of the immune TME has only been recognized over the past decade.^243^ As a result, immune-centered networks of PM-associated carcinogenesis remain substantially underexplored, representing a critical gap. Motivated by this imbalance, we propose a set of five interlocking hypotheses that collectively illuminate how PM progressively dismantles epithelial, stromal, and immune defenses to permit malignant transformation.

#### Hypothesis 1: Epigenetic-immunologic silencing of the early sensing machinery

Recent studies have shown that cGAS-STING and antigen-presentation pathways can be epigenetically silenced through epigenetic mechanisms in immune-evasive tumors.^280^ PM has been linked to altered methylation signatures,^167^ but no study has connected these epigenetic changes to the targeted suppression of innate immune pathways. We propose that chronic exposure to PM induces a coordinated epigenetic repression of cytosolic viral-sensing and antigen-presentation pathways, thereby establishing a state of early immune invisibility that permits PM-associated clones to persist and expand.

To evaluate this hypothesis, chronic PM exposure of primary human airway epithelial cells and co-cultured monocyte-derived dendritic cells should reveal locus-specific DNA methylation, H3K27 trimethylation, and reduced accessibility at STING and MHC-I-related genes, as measured by WGBS, ATAC-seq, and ChIP-seq.^281^ These changes should correlate with blunted IFN-β production, diminished cGAS-STING signaling, and impaired antigen presentation, and should be partially reversible with DNMT or EZH2 inhibitors or by exogenous STING agonists. In murine long-term aerosol exposure models, epithelial lesions arising under PM pressure are predicted to display suppressed STING/MHC-I expression and reduced T cell infiltration, whereas genetic or pharmacologic reactivation of these pathways should restore immune surveillance and restrain lesion progression. Human specimens from high-exposure regions should exhibit higher methylation at STING and antigen-presentation loci compared with low-exposure controls, along with correspondingly immune-cold microenvironments.

#### Hypothesis 2: PM-induced metabolic rewiring drives early adenosine-mediated immune suppression

Adenosine-driven immune suppression is recognized as a major route of immune escape in late-stage tumors,^280^ yet its involvement in environmental carcinogenesis remains unexplored. PM is known to induce glycolytic reprogramming in epithelial and myeloid cells,^183^^,^^250^ but no study has linked these shifts to the early collapse of immune danger signaling. We hypothesize that PM exposure shifts epithelial and immune-cell metabolism toward a glycolytic, lactate-rich state at pre-neoplastic stages, which promotes CD39/CD73-mediated extracellular depletion of ATP but accumulation of adenosine, thereby suppressing DC priming and T cell activation.

This hypothesis can be assessed by the following design. PM-treated epithelial and macrophage cultures should exhibit increased glycolytic rate, elevated lactate output, augmented CD39/CD73 expression, and a shift from ATP to adenosine dominance in the extracellular milieu. Disruption of CD39 or CD73 should restore ATP availability, enhance dendritic cell maturation, and increase T cell priming in co-culture models. In PM-exposed mouse lungs, early lesions are expected to show high CD39/CD73 expression and low immune infiltration, whereas blockade of the adenosine axis should restore immune activation and impair lesion progression. Human lung samples from individuals in high-PM regions should demonstrate similar metabolic-immunologic signatures, linking ambient exposures to functional immune suppression.

#### Hypothesis 3: PM-induced stromal conditioning creates a fibrotic-neutrophilic immune-exclusion barrier

Immune exclusion in solid cancers highlights cancer-associated fibroblasts (CAFs), neutrophils, and TGF-β-driven fibrosis as core structural barriers blocking cytotoxic T cell infiltration.^280^^,^^282^ PM exposure is well documented to elevate TGF-β and CXCL8 levels, promote fibroblast activation, and recruit neutrophils, but current studies have focused on injury and repair rather than the emergence of exclusionary TME.^171^^,^^172^^,^^283^^,^^284^ We propose that PM exposure drives a highly coordinated stromal reprogramming process that converts lung peritumoral regions into a fibrotic-neutrophilic exclusion zone, physically and functionally preventing cytotoxic lymphocyte entry.

This hypothesis can be examined through epithelial-fibroblast-neutrophil co-culture systems and *in vivo* perturbation experiments. Conditioned media from PM-treated epithelial or myeloid cells should induce fibroblast activation and matrix deposition, whereas blocking TGF-β or CXCL8 should attenuate these responses. Spatial-resolution analyses in PM-exposed mouse models and human tissues should reveal CAF and TAN enrichment at lesion margins and a reciprocal exclusion of CD8^+^ T cells, forming spatially segregated micro-domains. *In vivo* inhibition of TGF-β signaling or NET formation is expected to dismantle the exclusion architecture, restore lymphocyte infiltration, and decelerate PM-promoted lesion growth.

#### Hypothesis 4: PM-mediated impairment of HEV-TLS maturation as a catalyst for early immune escape

Emerging evidence shows that functional tertiary lymphoid structure (TLS) formation requires intact high endothelial venule (HEV) signaling and permissive stromal architecture, both of which are highly sensitive to oxidative and inflammatory injury,^285^ the two hallmarks of PM exposure as we discussed. We therefore hypothesize that PM-triggered endothelial oxidative injury and pro-angiogenic/VEGF-biased signaling lead to downregulation or phenotypic alteration of HEV-specific adhesion and chemokine cues, while concomitant TGF-β-mediated fibroblast activation and extracellular matrix deposition form a stromal barrier that prevents TLS assembly and germinal-center formation. The combined effect is a local failure of adaptive immune recruitment and *in situ* antigen presentation.

This hypothesis can be tested across *in vitro*, *in vivo*, and human-translational platforms. *In vitro*, primary human lung microvascular endothelial cells and HEV-differentiation models exposed to PM should be assayed for oxidative stress, PNAd/GlyCAM1 expression, LTβR signaling, and CCL21 production, and for functional lymphocyte rolling/adhesion using flow-based assays. Concurrent co-culture of PM-conditioned epithelial/myeloid secretomes with lung fibroblasts will test fibroblast activation (α-SMA, collagen) and barrier properties. *In vivo*, chronic aerosol exposure in murine models should be evaluated for TLS frequency, maturation state, spatial relation to particle deposits and to fibrotic matrix. Intervention applying LTβR agonism or anti-TGFβ/anti-fibrotic therapy will assess rescue of HEV/TLS formation and consequent effects on immune infiltration and tumor incidence. Finally, human validation should compare lung resections or screening biopsies from high- versus low-PM regions for TLS density, HEV marker expression, stromal fibrosis, and correlations with ambient/personal exposure estimates and early lesion clonality.

#### Hypothesis 5: The myeloid immunosuppression and ferroptosis hypothesis

Myeloid-derived suppressor cells (MDSCs) are known to accumulate in lung cancer and their ferroptosis in tumor is able to generate oxidized phospholipids, which suppress immune response.^286^ Given that PM2.5 can trigger ferroptosis and lipid peroxidation,^287^^,^^288^ we then propose that PM2.5 exposure induces ferroptosis in lung MDCS themselves, releasing the lipid peroxidation products to act as potent mediators of CD8+T cell effector function.

Validating this hypothesis could involve the following processes. First, we would confirm that PM2.5 exposure directly induces ferroptosis in MDSCs, by measuring indicators of lipid peroxidation (via C11-BODIPY staining), depletion of GPX4, and morphological changes in MDSCs isolated from PM-exposed mice or treated *in vitro*. Second, collect the conditioned medium from these ferroptotic MDSCs and apply it to activated T cells. The expected result is a significant suppression of T cell proliferation (measured by CFSE dilution) and IFN-γ production (by ELISA or flow cytometry), which should be rescued by pre-treating the MDSCs with ferroptosis inhibitors or by scavenging oxidized lipids in the medium. Finally, *in vivo* validation would involve treating mice with ferrostatin-1 and assessing whether the reduction in MDSC ferroptosis correlates with restored T cell function and controlled tumor growth, even if the number of tumor-infiltrating T cells remains unchanged.

In summary, the first two hypotheses interrogate the earliest phase of the host-particle interaction, where PM perturbs the immune-epithelial interface before any morphologic abnormality is detectable. Hypothesis 1 proposes that PM induces epigenetic-immunologic silencing of innate sensing machinery. Hypothesis 2 complements this by suggesting that PM-driven adenosine enrichment enforces an early metabolic immunosuppressive state that enables damaged epithelial clones to persist. The next two hypotheses examine how these early vulnerabilities propagate to reshape the lung ecosystem. Hypothesis 3 posits that PM establishes a fibrotic, inflammatory immune-exclusion barrier and hypothesis 4 proposes that PM disrupts the maturation of HEV and TLS. Finally, hypothesis 5 addresses the culminating stage of PM-induced immune breakdown. Together, these five hypotheses will prove how PM subverts both the innate and adaptive arms of immunity to promote lung cancer evolution.

## Bridging PM-associated hallmarks and clinical practice

**Figure 7:**
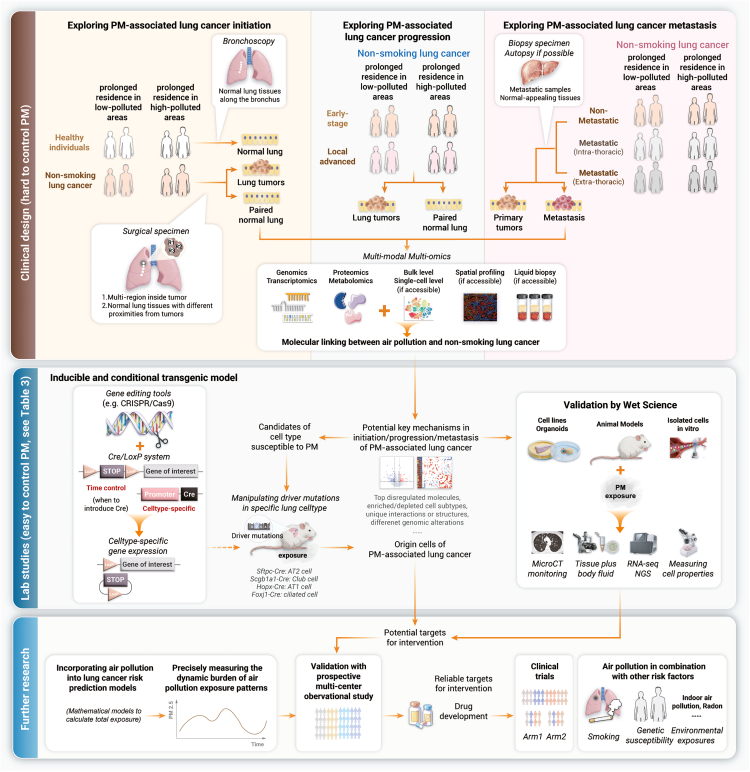
Recommendations for future research design on PM-associated lung cancer Future studies on air pollution-associated lung cancer should embrace a comprehensive approach, amalgamating clinical and laboratory investigations. This entails procuring tissue samples from lung cancer patients across various stages of progression, residing in regions with distinct pollution levels, ideally juxtaposed with normal tissue samples for comparison. Subsequently, employing multi-omics techniques, researchers should delve into the potential mechanisms of PM at different phases of lung cancer evolution. To identify potential targets, rigorous validation through *ex vivo* experiments is imperative, complemented by diverse analytical methodologies. Concurrently, the quest for the cells of origin can be pursued through genetically engineered mice. Once reliable targets have been discerned, the development of target-specific drugs is warranted, followed by rigorous clinical trials to authenticate their efficacy. This comprehensive approach holds promise for fostering interventions and treatments for air pollution-associated lung cancer, furnishing a robust foundation for future endeavors.

Understanding the layered architecture of PM-associated carcinogenesis enables translation into clinical research, practice settings, and public health policies (Figure 7). This section tries to map these mechanistic vulnerabilities onto actionable interventions.

### PM-specific biomarkers in lung cancer patients

Lacking clinical sample profiling combined with exposure measurements, molecular features of populations chronically exposed to PM remain elusive both for healthy individuals and lung cancer patients. While some research has explored the impact of PM on serum, circulating cells, urine, and lung tissue, they often focus on acute exposure.^289^^,^^290^^,^^291^^,^^292^^,^^293^^,^^294^^,^^295^^,^^296^^,^^297^^,^^298^ Few studies have investigated the lung cancer microenvironment in regions with high versus low pollution levels.^60^^,^^147^ Ideal protocols start with investigating the non-smoking populations under varying levels of PM exposure, including lung cancer at different stages and healthy individuals quantitatively assessing their exposure to PM. The next step involves multi-omics analyses of samples from primary tumors, metastatic tumors, paired healthy tissues, and blood to reconstruct the multi-layers of PM-driven PM-specific molecular trajectories. These analyses include but are not limited to genomic sequencing for identifying mutation spectra and clonality patterns enriched in chronically exposed populations, epigenomic profiling to reveal long-lasting chromatin alterations and methylation signatures reflective of cumulative PM burden, transcriptomics and proteomics to characterize pathway-level reprogramming associated with PM hallmarks, metabolomics to provide insight into redox imbalance and xenobiotic biotransformation induced by PM chemical constituents, spatiomics and single-cell transcriptomics to resolve how PM exposure reshapes the architecture of immune-stromal niches within the lung. Subsequently, identified targets should be validated using animal or cellular models to ensure their reliability.

From the translational aspect, heavier air pollution shortens the survival in lung cancer,^101^ necessitating the inclusion of PM itself in prognostic stratification. Ongoing trials are also advised to incorporate PM exposure as a variable regarding the adverse influence of PM on treatment outcomes.^299^ Beyond the numeric pollution level as one prognostic factor, PM exposure generates 12 relevant hallmarks as we discussed before. Hence, future research should focus on developing prognostic and predictive biomarkers of PM-associated lung cancer, which holds promise for novel avenues for relapse monitoring and treating prediction. Following identification, it is further possible to develop accessible tests for clinical settings.

Inherent limitations exist in establishing direct causality in human environmental oncology. Unlike controlled laboratory settings, human studies usually fail to isolate PM exposure as the sole causative factor. A primary challenge is confounding, as individuals exposed to high levels of air pollution often differ in numerous other ways, such as socioeconomic status, occupational hazards, and lifestyle factors, all of which may independently influence lung cancer risk. It is advised to adopt statistical models designed for omics analysis to adjust for these variables.^300^^,^^301^ Additionally, direct experimental testing of carcinogenicity in humans is ethically impermissible. Consequently, causal inference must rely on the triangulation of evidence from multiple facets. This includes consistency across independent epidemiological studies, biological plausibility established through *in vitro* and *in vivo* experiments, and coherence with known pathological mechanisms. Population experiments involving air pollution reduction policies will be critical to further strengthening causal claims.

### Integrating PM into lung cancer screening and prevention

Current screening trials of lung cancer primarily target smokers or individuals with high occupational exposures.^302^^,^^303^ These programs neglect non-smokers in air-polluted areas^304^ and may not suit traditionally low-risk groups. Meanwhile, PM has hardly been 0included in validated models predicting lung cancer risk,^305^^,^^306^ which ought to be considered in future screening protocols. In addition, it is crucial to modify the screening models according to regional pollutant levels, considering the complex non-linear observed in current evidence.^78^^,^^79^

This leaves another question, lacking measurement of individual exposure to inhalable PM. Early studies relied on fixed monitoring station,^81^^,^^82^^,^^84^^,^^107^^,^^307^ limited in spatial coverage and individual quantification. Satellite-based remote sensing^80^^,^^85^^,^^112^ and the land use regression (LUR) models combining geographic information systems (GIS) with pollution data have provided records of higher quality.^42^^,^^89^^,^^308^ More precisely, developing wearable sensors or mobile apps that track PM concentration in real time, and incorporating this information into medical records is needed.

To reduce the PM levels, pollutant control strategies^309^^,^^310^ have yielded significant health benefits in this century.^311^^,^^312^ While decreasing overall PM concentrations remains a key objective, PM from different sources need tailored emission standards given their non-uniform biological impacts.^309^^,^^310^^,^^313^ For example, PM’s oxidative potential is more relevant to anthropogenic sources such as vehicle emissions and secondary organic aerosols than agriculture.^314^ For individual prevention, pharmaceutical interventions are largely constrained considering the enduring impact of air pollution. Instead, dietary interventions seem more feasible. Supplementing vitamin B has been shown to neutralize changes in DNA methylation levels induced by PM2.5.^315^ And L-arginine may mitigate the oxidative effects of PM exposure.^316^^,^^317^ The direct protection of nutrient intake requires further investigation in future studies.

## Conclusion and perspectives

Over decades, research on PM-associated lung cancer has evolved from a largely overlooked topic in the mid-20th century to a rapidly expanding field. In this review, we introduce the novel “Hallmarks of PM-associated lung cancer” as a unifying conceptual framework that captures the unique biological mechanisms spanning lung cancer evolution driven by PM. It is revealed that PM functions not merely as a mutagen but influences far beyond the respiratory tract. Its carcinogenicity on lung epithelial cells forms the notable hallmarks encompassing direct genotoxic damage, epigenetic dysregulation, stem-like reprogramming, and metabolic rewiring. Simultaneously, PM actively sculpts a tumor-supportive ecosystem by polarizing immune cells toward immunosuppression, stimulating angiogenesis, and inducing pre-metastatic niches in distant organs. Chronic inflammation serves as an interconnected hub mediating these pro-tumorigenic effects. Consequently, lung cancers arising under chronic PM exposure constitute a distinct entity. These PM-associated hallmarks offer a systematic lens for interpreting diverse findings and identifying mechanistic vulnerabilities.

Despite significant advances, several critical gaps remain. Current experimental models often fail to recapitulate the exposure patterns observed in real-world settings and often lack the complexity of the human immune system. Standardization in PM characterization, exposure protocols, and model systems remains elusive. Bridging the chasm between mechanistic insights and clinical application is paramount. This requires the robust integration of individually quantified, cumulative PM exposure metrics into lung cancer risk prediction models and screening programs, particularly to identify high-risk non-smokers currently overlooked by traditional criteria. Concurrently, an urgent need exists to identify and validate PM-specific molecular biomarkers through multi-dimensional clinical profiling of clinical samples from individuals with well-documented exposure histories, for improved prognostic stratification, monitoring of disease progression and therapy response, and the development of targeted preventive and therapeutic strategies. Ultimately, mitigating the global burden of PM-associated lung cancer, particularly within susceptible non-smoking groups residing in highly contaminated areas, demands not only innovative research but also the rigorous implementation and enforcement of source-specific emission controls targeting the most toxic PM components, alongside public health initiatives focused on awareness and feasible protective interventions. Through concerted scientific, clinical, and public efforts, addressing the sophisticated interplay betwixt air pollution and lung cancer biology will not only provide novel insights into environmental carcinogenesis but also offers a unique opportunity to advance precision prevention and intervention strategies for populations living under the threat of air pollution.

## Funding and acknowledgments

This work was supported by the 10.13039/501100001809National Natural Science Foundation of China (82525052, 82373416, 82388102, 82450111, and 92259303); the 10.13039/501100005150Chinese Academy of Medical Sciences (2021RU002); 10.13039/501100004826Beijing Natural Science Foundation (Z240013, L234002, L222021, and QY23070); the Noncommunicable Chronic Diseases-National Science and Technology Major Project (2023ZD0501900 and 2023ZD0501902); the Beijing Research Ward Excellence Program (BRWEP2024W034080200 and BRWEP2024W034080204); the Science, Technology &Innovation Project of Xiongan New Area (2023XAGG0071); the 10.13039/501100003345CAMS Medical and Health Science and Technology Innovation Project (2021-I2M-5-002); 10.13039/501100007937Peking University Clinical Scientist Training Program, supported by the Fundamental Research Funds for the Central Universities (BMU2025PYJH001); 10.13039/501100015083Peking University People's Hospital Research and Development Funds (RZG2024-02). The funders had no role in study design, data collection and analysis, decision to publish, or preparation of the manuscript.

## Author contributions

K.C., X.Y., and C.W. conceived and organized the review. X.Y., X.L., and C.W. wrote the manuscript. Y.W., R.J., E.L., F.W., and L.W. participated in manuscript revision and proofreading. H.L., L.Q., Z.Z., Q.J., O.C., D.C., J.S., F.Y., J.W., K.Z., C.-Y.C., and F.K. edited the manuscript. D.C., J.W., and K.C. supervised the manuscript editing and revision. All authors contributed to the manuscript and approved the final version.

## Declaration of interests

The authors declare no competing interests.

## Supplemental information

Data S1. Tables S1 and S3

Document S2. Article plus supplemental information

## References

[1] Wang J. (2024). CACA guidelines for holistic integrative management of lung cancer. Holist. Integr. Oncol..

[2] Sung H., Ferlay J., Siegel R.L. (2021). Global Cancer Statistics 2020: GLOBOCAN Estimates of Incidence and Mortality Worldwide for 36 Cancers in 185 Countries. CA Cancer J. Clin..

[3] Islami F., Goding Sauer A., Miller K.D. (2018). Proportion and number of cancer cases and deaths attributable to potentially modifiable risk factors in the United States. CA Cancer J. Clin..

[4] Tan N., Li Y., Ying J. (2024). Histological transformation in lung adenocarcinoma: Insights of mechanisms and therapeutic windows. J. Transl. Int. Med..

[5] Loomis D., Grosse Y., Lauby-Secretan B. (2013). The carcinogenicity of outdoor air pollution. Lancet Oncol..

[6] Berg C.D., Schiller J.H., Boffetta P. (2023). Air Pollution and Lung Cancer: A Review by International Association for the Study of Lung Cancer Early Detection and Screening Committee. J. Thorac. Oncol..

[7] Xue Y., Wang L., Zhang Y. (2022). Air pollution: A culprit of lung cancer. J. Hazard. Mater..

[8] GBD 2021 Risk Factors Collaborators (2024). Global burden and strength of evidence for 88 risk factors in 204 countries and 811 subnational locations, 1990-2021: a systematic analysis for the Global Burden of Disease Study 2021. Lancet.

[9] Díaz-Gay M., Zhang T., Hoang P.H. (2025). The mutagenic forces shaping the genomes of lung cancer in never smokers. Nature.

[10] Bonetta S., Bonetta S., Schilirò T. (2018). Mutagenic and genotoxic effects induced by PM0.5 of different Italian towns in human cells and bacteria: The MAPEC_LIFE study. Environ. Pollut..

[11] Balmain A. (2020). The critical roles of somatic mutations and environmental tumor-promoting agents in cancer risk. Nat. Genet..

[12] Hill W., Lim E.L., Weeden C.E. (2023). Lung adenocarcinoma promotion by air pollutants. Nature.

[13] Kerpel-Fronius A., Tammemägi M., Cavic M. (2022). Screening for Lung Cancer in Individuals Who Never Smoked: An International Association for the Study of Lung Cancer Early Detection and Screening Committee Report. J. Thorac. Oncol..

[14] Myers R., Brauer M., Dummer T. (2021). High-Ambient Air Pollution Exposure Among Never Smokers Versus Ever Smokers With Lung Cancer. J. Thorac. Oncol..

[15] Liu C., Chen R., Sera F. (2019). Ambient Particulate Air Pollution and Daily Mortality in 652 Cities. N. Engl. J. Med..

[16] Yue H., Yun Y., Gao R. (2015). Winter Polycyclic Aromatic Hydrocarbon-Bound Particulate Matter from Peri-urban North China Promotes Lung Cancer Cell Metastasis. Environ. Sci. Technol..

[17] Blenkinsopp W.K. (1968). Particle accumulation in the lung as a possible factor in the aetiology of lung cancer. J. Pathol. Bacteriol..

[18] Kotin P. (1956). The role of atmospheric pollution in the pathogenesis of pulmonary cancer; a review. Cancer Res..

[19] Dockery D.W., Pope C.A., Xu X. (1993). An association between air pollution and mortality in six U.S. cities. N. Engl. J. Med..

[20] Pope C.A., Burnett R.T., Thun M.J. (2002). Lung cancer, cardiopulmonary mortality, and long-term exposure to fine particulate air pollution. JAMA.

[21] Pope C.A., Thun M.J., Namboodiri M.M. (1995). Particulate air pollution as a predictor of mortality in a prospective study of U.S. adults. Am. J. Respir. Crit. Care Med..

[22] GBD 2021 HAP Collaborators (2025). national burden of household air pollution, 1990-2021: a systematic analysis for the Global Burden of Disease Study 2021. Lancet (London, England).

[23] Annesi-Maesano I., Forastiere F., Kunzli N. (2007). Particulate matter, science and EU policy. Eur. Respir. J..

[24] Izzotti A., Camoirano A., D'Agostini F. (1996). Biomarker alterations produced in rat lung by intratracheal instillations of air particulate extracts and chemoprevention with oral N-acetylcysteine. Cancer Res..

[25] Timblin C., BeruBe K., Churg A. (1998). Ambient particulate matter causes activation of the c-jun kinase/stress-activated protein kinase cascade and DNA synthesis in lung epithelial cells. Cancer Res..

[26] Turner M.C., Andersen Z.J., Baccarelli A. (2020). Outdoor air pollution and cancer: An overview of the current evidence and public health recommendations. CA Cancer J. Clin..

[27] Hamanaka R.B., Mutlu G.M. (2025). Particulate matter air pollution: effects on the respiratory system. J. Clin. Investig..

[28] Sun J., Yu J., Niu X. (2023). Solid fuel derived PM2.5 induced oxidative stress and according cytotoxicity in A549 cells: The evidence and potential neutralization by green tea. Environ. Int..

[29] Cheung K., Daher N., Kam W. (2011). Spatial and temporal variation of chemical composition and mass closure of ambient coarse particulate matter (PM10–2.5) in the Los Angeles area. Atmos. Environ..

[30] Luo F., Guo H., Yu H. (2021). PM2.5 organic extract mediates inflammation through the ERbeta pathway to contribute to lung carcinogenesis in vitro and vivo. Chemosphere.

[31] Radan M., Dianat M., Badavi M. (2019). In vivo and in vitro evidence for the involvement of Nrf2-antioxidant response element signaling pathway in the inflammation and oxidative stress induced by particulate matter (PM10): the effective role of gallic acid. Free Radic. Res..

[32] Wang T., Rovira J., Sierra J. (2021). Characterization of airborne particles and cytotoxicity to a human lung cancer cell line in Guangzhou, China. Environ. Res..

[33] Pope C.A. (2000). Epidemiology of fine particulate air pollution and human health: biologic mechanisms and who's at risk?. Environ. Health Perspect..

[34] Zou Y., Wu Y., Wang Y. (2017). Physicochemical properties, in vitro cytotoxic and genotoxic effects of PM(1.0) and PM(2.5) from Shanghai, China. Environ. Sci. Pollut. Res. Int..

[35] Du W., Yun X., Chen Y. (2020). PAHs emissions from residential biomass burning in real-world cooking stoves in rural China. Environ. Pollut..

[36] Wang T., Li B., Huang T. (2023). Long-term spatiotemporal variation and lung cancer risk of atmospheric polycyclic aromatic hydrocarbons (PAHs) in the Yangtze River Delta, China. Environ. Geochem. Health.

[37] Guo H., Zhang Z., Wang H. (2021). Oxidative stress and inflammatory effects in human lung epithelial A549 cells induced by phenanthrene, fluorene, and their binary mixture. Environ. Toxicol..

[38] Niu X., Jones T., BéruBé K. (2021). The oxidative capacity of indoor source combustion derived particulate matter and resulting respiratory toxicity. Sci. Total Environ..

[39] Song Y., Li R., Zhang Y. (2019). Mass spectrometry-based metabolomics reveals the mechanism of ambient fine particulate matter and its components on energy metabolic reprogramming in BEAS-2B cells. Sci. Total Environ..

[40] Barzgar F., Sadeghi-Mohammadi S., Aftabi Y. (2023). Oxidative stress indices induced by industrial and urban PM(2.5)-bound metals in A549 cells. Sci. Total Environ..

[41] Lovera-Leroux M., Crobeddu B., Kassis N. (2015). The iron component of particulate matter is antiapoptotic: A clue to the development of lung cancer after exposure to atmospheric pollutants?. Biochimie.

[42] Raaschou-Nielsen O., Andersen Z.J., Beelen R. (2013). Air pollution and lung cancer incidence in 17 European cohorts: prospective analyses from the European Study of Cohorts for Air Pollution Effects (ESCAPE). Lancet Oncol..

[43] Raaschou-Nielsen O., Beelen R., Wang M. (2016). Particulate matter air pollution components and risk for lung cancer. Environ. Int..

[44] Stafoggia M., Oftedal B., Chen J. (2022). Long-term exposure to low ambient air pollution concentrations and mortality among 28 million people: results from seven large European cohorts within the ELAPSE project. Lancet Planet. Health.

[45] So R., Chen J., Stafoggia M. (2023). Long-term exposure to elemental components of fine particulate matter and all-natural and cause-specific mortality in a Danish nationwide administrative cohort study. Environ. Res..

[46] Lequy E., Leblond S., Siemiatycki J. (2023). Long-term exposure to airborne metals and risk of cancer in the French cohort Gazel. Environ. Int..

[47] Raaschou-Nielsen O., Antonsen S., Agerbo E. (2023). PM(2.5) air pollution components and mortality in Denmark. Environ. Int..

[48] Zeng Q., Li G., Zhao L. (2015). Characteristics of the Exposure-Response Relationship of Particulate Matter and Mortality: A Time Series Analysis of 7 Cities in China. J. Occup. Environ. Med..

[49] Shen H., Huang Y., Wang R. (2013). Global atmospheric emissions of polycyclic aromatic hydrocarbons from 1960 to 2008 and future predictions. Environ. Sci. Technol..

[50] Madaniyazi L., Guo Y., Chen R. (2016). Predicting exposure-response associations of ambient particulate matter with mortality in 73 Chinese cities. Environ. Pollut..

[51] Becker S., Dailey L., Soukup J.M. (2005). TLR-2 is involved in airway epithelial cell response to air pollution particles. Toxicol. Appl. Pharmacol..

[52] Pan S.Y., Chi K.H., Wang Y.C. (2022). Sub-toxic events induced by truck speed-facilitated PM(2.5) and its counteraction by epigallocatechin-3-gallate in A549 human lung cells. Sci. Rep..

[53] Borgie M., Ledoux F., Verdin A. (2015). Genotoxic and epigenotoxic effects of fine particulate matter from rural and urban sites in Lebanon on human bronchial epithelial cells. Environ. Res..

[54] Ndong Ba A., Cazier F., Verdin A. (2019). Physico-chemical characterization and in vitro inflammatory and oxidative potency of atmospheric particles collected in Dakar city's (Senegal). Environ. Pollut..

[55] Zhang S., Zhang J., Guo D. (2021). Biotoxic effects and gene expression regulation of urban PM(2.5) in southwestern China. Sci. Total Environ..

[56] Chafe Z.A., Brauer M., Klimont Z. (2014). Household cooking with solid fuels contributes to ambient PM2.5 air pollution and the burden of disease. Environ. Health Perspect..

[57] Mondal N.K., Saha H., Mukherjee B. (2018). Inflammation, oxidative stress, and higher expression levels of Nrf2 and NQO1 proteins in the airways of women chronically exposed to biomass fuel smoke. Mol. Cell. Biochem..

[58] Sun J., Shen Z., Zeng Y. (2018). Characterization and cytotoxicity of PAHs in PM(2.5) emitted from residential solid fuel burning in the Guanzhong Plain, China. Environ. Pollut..

[59] Dinh N.T.H., Lee J., Lee J. (2020). Indoor dust extracellular vesicles promote cancer lung metastasis by inducing tumour necrosis factor-alpha. J. Extracell. Vesicles.

[60] Zhang H., Liu C., Wang S. (2024). Proteogenomic analysis of air-pollution-associated lung cancer reveals prevention and therapeutic opportunities. eLife.

[61] Tomašek I., Horwell C.J., Damby D.E. (2016). Combined exposure of diesel exhaust particles and respirable Soufriere Hills volcanic ash causes a (pro-)inflammatory response in an in vitro multicellular epithelial tissue barrier model. Part. Fibre Toxicol..

[62] Zarcone M.C., Duistermaat E., van Schadewijk A. (2016). Cellular response of mucociliary differentiated primary bronchial epithelial cells to diesel exhaust. Am. J. Physiol. Lung Cell. Mol. Physiol..

[63] Bayram H., Ito K., Issa R. (2006). Regulation of human lung epithelial cell numbers by diesel exhaust particles. Eur. Respir. J..

[64] Li W., Liu T., Xiong Y. (2018). Diesel exhaust particle promotes tumor lung metastasis via the induction of BLT1-mediated neutrophilic lung inflammation. Cytokine.

[65] Chen S., Chen L., Ye L. (2022). PP2A-mTOR-p70S6K/4E-BP1 axis regulates M1 polarization of pulmonary macrophages and promotes ambient particulate matter induced mouse lung injury. J. Hazard. Mater..

[66] Ma Q.Y., Huang D.Y., Zhang H.J. (2017). Exposure to particulate matter 2.5 (PM2.5) induced macrophage-dependent inflammation, characterized by increased Th1/Th17 cytokine secretion and cytotoxicity. Int. Immunopharmacol..

[67] Hou T., Liao J., Zhang C. (2018). Elevated expression of miR-146, miR-139 and miR-340 involved in regulating Th1/Th2 balance with acute exposure of fine particulate matter in mice. Int. Immunopharmacol..

[68] Meldrum K., Gant T.W., Leonard M.O. (2017). Diesel exhaust particulate associated chemicals attenuate expression of CXCL10 in human primary bronchial epithelial cells. Toxicol. Vitro.

[69] Hu W., Downward G.S., Reiss B. (2014). Personal and indoor PM2.5 exposure from burning solid fuels in vented and unvented stoves in a rural region of China with a high incidence of lung cancer. Environ. Sci. Technol..

[70] Hosgood H.D., Song M., Hsiung C.A. (2015). Interactions between household air pollution and GWAS-identified lung cancer susceptibility markers in the Female Lung Cancer Consortium in Asia (FLCCA). Hum. Genet..

[71] Yang W.S., Zhao H., Wang X. (2016). An evidence-based assessment for the association between long-term exposure to outdoor air pollution and the risk of lung cancer. Eur. J. Cancer Prev..

[72] Cui P., Huang Y., Han J. (2015). Ambient particulate matter and lung cancer incidence and mortality: a meta-analysis of prospective studies. Eur. J. Publ. Health.

[73] Ciabattini M., Rizzello E., Lucaroni F. (2021). Systematic review and meta-analysis of recent high-quality studies on exposure to particulate matter and risk of lung cancer. Environ. Res..

[74] Arif I., Adams M.D., Johnson M.T.J. (2024). A meta-analysis of the carcinogenic effects of particulate matter and polycyclic aromatic hydrocarbons. Environ. Pollut..

[75] Zhang T., Mao W., Gao J. (2022). The effects of PM2.5 on lung cancer-related mortality in different regions and races: A systematic review and meta-analysis of cohort studies. Air Qual. Atmos. Health.

[76] Hamra G.B., Guha N., Cohen A. (2014). Outdoor particulate matter exposure and lung cancer: a systematic review and meta-analysis. Environ. Health Perspect..

[77] Yu P., Guo S., Xu R. (2021). Cohort studies of long-term exposure to outdoor particulate matter and risks of cancer: A systematic review and meta-analysis. Innovation.

[78] Li W., Tian A., Shi Y. (2023). Associations of long-term fine particulate matter exposure with all-cause and cause-specific mortality: results from the ChinaHEART project. Lancet Reg. Health West. Pac..

[79] Liu H., Qi J., Ren W. (2025). Disparities in trends and drivers of the burden of tracheal, bronchus, and lung cancer among Chinese population during 1990-2021: a systematic analysis for the global burden of disease study 2021. Sci. Bull..

[80] Li J., Lu X., Liu F. (2020). Chronic Effects of High Fine Particulate Matter Exposure on Lung Cancer in China. Am. J. Respir. Crit. Care Med..

[81] Gharibvand L., Shavlik D., Ghamsary M. (2017). The Association between Ambient Fine Particulate Air Pollution and Lung Cancer Incidence: Results from the AHSMOG-2 Study. Environ. Health Perspect..

[82] Lepeule J., Laden F., Dockery D. (2012). Chronic exposure to fine particles and mortality: an extended follow-up of the Harvard Six Cities study from 1974 to 2009. Environ. Health Perspect..

[83] Turner M.C., Krewski D., Pope C.A. (2011). Long-term ambient fine particulate matter air pollution and lung cancer in a large cohort of never-smokers. Am. J. Respir. Crit. Care Med..

[84] Fischer P.H., Marra M., Ameling C.B. (2015). Air Pollution and Mortality in Seven Million Adults: The Dutch Environmental Longitudinal Study (DUELS). Environ. Health Perspect..

[85] Cakmak S., Hebbern C., Pinault L. (2018). Associations between long-term PM(2.5) and ozone exposure and mortality in the Canadian Census Health and Environment Cohort (CANCHEC), by spatial synoptic classification zone. Environ. Int..

[86] Krewski D., Jerrett M., Burnett R.T. (2009). Extended follow-up and spatial analysis of the American Cancer Society study linking particulate air pollution and mortality. Res. Rep. Health Eff. Inst..

[87] Pond Z.A., Saha P.K., Coleman C.J. (2022). Mortality risk and long-term exposure to ultrafine particles and primary fine particle components in a national U.S. Cohort. Environ. Int..

[88] Jones R.R., Fisher J.A., Hasheminassab S. (2024). Outdoor Ultrafine Particulate Matter and Risk of Lung Cancer in Southern California. Am. J. Respir. Crit. Care Med..

[89] Bouma F., Janssen N.A., Wesseling J. (2023). Long-term exposure to ultrafine particles and natural and cause-specific mortality. Environ. Int..

[90] Weichenthal S., Bai L., Hatzopoulou M. (2017). Long-term exposure to ambient ultrafine particles and respiratory disease incidence in in Toronto, Canada: a cohort study. Environ. Health.

[91] Olsson A.C., Gustavsson P., Kromhout H. (2011). Exposure to diesel motor exhaust and lung cancer risk in a pooled analysis from case-control studies in Europe and Canada. Am. J. Respir. Crit. Care Med..

[92] Silverman D.T., Bassig B.A., Lubin J. (2023). The Diesel Exhaust in Miners Study (DEMS) II: Temporal Factors Related to Diesel Exhaust Exposure and Lung Cancer Mortality in the Nested Case-Control Study. Environ. Health Perspect..

[93] Ge C., Peters S., Olsson A. (2020). Diesel Engine Exhaust Exposure, Smoking, and Lung Cancer Subtype Risks. A Pooled Exposure-Response Analysis of 14 Case-Control Studies. Am. J. Respir. Crit. Care Med..

[94] Parent M.E., Rousseau M.C., Boffetta P. (2007). Exposure to diesel and gasoline engine emissions and the risk of lung cancer. Am. J. Epidemiol..

[95] Ilar A., Plato N., Lewné M. (2017). Occupational exposure to diesel motor exhaust and risk of lung cancer by histological subtype: a population-based case-control study in Swedish men. Eur. J. Epidemiol..

[96] Bell M.L., Dominici F., Ebisu K. (2007). Spatial and temporal variation in PM(2.5) chemical composition in the United States for health effects studies. Environ. Health Perspect..

[97] Li C., van Donkelaar A., Hammer M.S. (2023). Reversal of trends in global fine particulate matter air pollution. Nat. Commun..

[98] McKeon T.P., Anil V., Penning T.M. (2022). Air pollution and lung cancer survival in Pennsylvania. Lung Cancer.

[99] Liu Y., Li D., Ren M. (2023). Effect of high-level PM(2.5) on survival in lung cancer: a multicenter cohort study from Hebei Province, China. Environ. Sci. Pollut. Res. Int..

[100] Kosanpipat B., Wongwut T., Norrasan N. (2024). Impact of PM2.5 exposure on mortality and tumor recurrence in resectable non-small cell lung carcinoma. Sci. Rep..

[101] Eckel S.P., Cockburn M., Shu Y.H. (2016). Air pollution affects lung cancer survival. Thorax.

[102] Liu C., Yang D., Liu Y. (2023). The effect of ambient PM(2.5) exposure on survival of lung cancer patients after lobectomy. Environ. Health.

[103] Yu P., Xu R., Wu Y. (2024). Cancer mortality risk from short-term PM(2.5) exposure and temporal variations in Brazil. J. Hazard. Mater..

[104] Ma C., Jung C.R., Nakayama S.F. (2023). Short-term association of air pollution with lung cancer mortality in Osaka, Japan. Environ. Res..

[105] Yang L., Wang N., Liu S. (2023). The PM(2.5) concentration reduction improves survival rate of lung cancer in Beijing. Sci. Total Environ..

[106] Liu C.S., Wei Y., Danesh Yazdi M. (2023). Long-term association of air pollution and incidence of lung cancer among older Americans: A national study in the Medicare cohort. Environ. Int..

[107] Pope C.A., Lefler J.S., Ezzati M. (2019). Mortality Risk and Fine Particulate Air Pollution in a Large, Representative Cohort of U.S. Adults. Environ. Health Perspect..

[108] Guo T., Chen S., Wang Y. (2024). Potential causal links of long-term air pollution with lung cancer incidence: From the perspectives of mortality and hospital admission in a large cohort study in southern China. Int. J. Cancer.

[109] Pun V.C., Kazemiparkouhi F., Manjourides J. (2017). Long-Term PM2.5 Exposure and Respiratory, Cancer, and Cardiovascular Mortality in Older US Adults. Am. J. Epidemiol..

[110] Tomczak A., Miller A.B., Weichenthal S.A. (2016). Long-term exposure to fine particulate matter air pollution and the risk of lung cancer among participants of the Canadian National Breast Screening Study. Int. J. Cancer.

[111] Abbey D.E., Nishino N., McDonnell W.F. (1999). Long-term inhalable particles and other air pollutants related to mortality in nonsmokers. Am. J. Respir. Crit. Care Med..

[112] Puett R.C., Hart J.E., Yanosky J.D. (2014). Particulate matter air pollution exposure, distance to road, and incident lung cancer in the nurses' health study cohort. Environ. Health Perspect..

[113] Yin P., Brauer M., Cohen A. (2017). Long-term Fine Particulate Matter Exposure and Nonaccidental and Cause-specific Mortality in a Large National Cohort of Chinese Men. Environ. Health Perspect..

[114] Siddharthan T., Grigsby M.R., Goodman D. (2018). Association between Household Air Pollution Exposure and Chronic Obstructive Pulmonary Disease Outcomes in 13 Low- and Middle-Income Country Settings. Am. J. Respir. Crit. Care Med..

[115] Olsson A., Guha N., Bouaoun L. (2022). Occupational Exposure to Polycyclic Aromatic Hydrocarbons and Lung Cancer Risk: Results from a Pooled Analysis of Case-Control Studies (SYNERGY). Cancer Epidemiol. Biomarkers Prev..

[116] Turner M.C., Cohen A., Jerrett M. (2014). Interactions between cigarette smoking and fine particulate matter in the Risk of Lung Cancer Mortality in Cancer Prevention Study II. Am. J. Epidemiol..

[117] Huang Y., Zhu M., Ji M. (2021). Air Pollution, Genetic Factors, and the Risk of Lung Cancer: A Prospective Study in the UK Biobank. Am. J. Respir. Crit. Care Med..

[118] Wang X., Wang T., Hua J. (2023). Histological types of lung cancer attributable to fine particulate, smoking, and genetic susceptibility. Sci. Total Environ..

[119] Liang H., Zhou X., Zhu Y. (2023). Association of outdoor air pollution, lifestyle, genetic factors with the risk of lung cancer: A prospective cohort study. Environ. Res..

[120] Li W., Wang W. (2024). Causal effects of exposure to ambient air pollution on cancer risk: Insights from genetic evidence. Sci. Total Environ..

[121] Siegel R.L., Giaquinto A.N., Jemal A. (2024). Cancer statistics, 2024. CA Cancer J. Clin..

[122] Kang H.R., Cho J.Y., Lee S.H. (2019). Role of Low-Dose Computerized Tomography in Lung Cancer Screening among Never-Smokers. J. Thorac. Oncol..

[123] Chang G.C., Chiu C.H., Yu C.J. (2024). Low-dose CT screening among never-smokers with or without a family history of lung cancer in Taiwan: a prospective cohort study. Lancet Respir. Med..

[124] Yi C.A., Lee K.S., Shin M.H. (2015). Low-dose CT screening in an Asian population with diverse risk for lung cancer: A retrospective cohort study. Eur. Radiol..

[125] Li Y., Du Y., Huang Y. (2021). Community-based lung cancer screening by low-dose computed tomography in China: first round results and a meta-analysis. Eur. J. Radiol..

[126] Zhang Y., Jheon S., Li H. (2020). Results of low-dose computed tomography as a regular health examination among Chinese hospital employees. J. Thorac. Cardiovasc. Surg..

[127] Wu F.Z., Huang Y.L., Wu C.C. (2016). Assessment of Selection Criteria for Low-Dose Lung Screening CT Among Asian Ethnic Groups in Taiwan: From Mass Screening to Specific Risk-Based Screening for Non-Smoker Lung Cancer. Clin. Lung Cancer.

[128] Liu X., Liang M., Wang Y. (2011). The outcome differences of CT screening for lung cancer pre and post following an algorithm in Zhuhai, China. Lung Cancer.

[129] Shan W., Chen Z., Wei D. (2021). Lung cancer screening with low-dose computed tomography at a tertiary hospital in Anhui, China and secondary analysis of trial data. Br. J. Radiol..

[130] World Health Organization (2021). WHO global air quality guidelines: particulate matter (PM2.5 and PM10), ozone, nitrogen dioxide, sulfur dioxide and carbon monoxide. https://www.who.int/publications/i/item/9789240034228/.

[131] Horeweg N., Scholten E.T., de Jong P.A. (2014). Detection of lung cancer through low-dose CT screening (NELSON): a prespecified analysis of screening test performance and interval cancers. Lancet Oncol..

[132] Lin W.C., Shie R.H., Yuan T.H. (2024). A nationwide case-referent study on elevated risks of adenocarcinoma lung cancer by long-term PM(2.5) exposure in Taiwan since 1997. Environ. Res..

[133] Hystad P., Demers P.A., Johnson K.C. (2013). Long-term residential exposure to air pollution and lung cancer risk. Epidemiology.

[134] Gharibvand L., Lawrence Beeson W., Shavlik D. (2017). The association between ambient fine particulate matter and incident adenocarcinoma subtype of lung cancer. Environ. Health.

[135] Tseng C.H., Tsuang B.J., Chiang C.J. (2019). The Relationship Between Air Pollution and Lung Cancer in Nonsmokers in Taiwan. J. Thorac. Oncol..

[136] Li F., Xiang B., Jin Y. (2019). Dysregulation of lipid metabolism induced by airway exposure to polycyclic aromatic hydrocarbons in C57BL/6 mice. Environ. Pollut..

[137] Denissenko M.F., Pao A., Tang M. (1996). Preferential formation of benzo[a]pyrene adducts at lung cancer mutational hotspots in P53. Science.

[138] Petruzzelli S., Celi A., Pulerà N. (1998). Serum antibodies to benzo(a)pyrene diol epoxide-DNA adducts in the general population: effects of air pollution, tobacco smoking, and family history of lung diseases. Cancer Res..

[139] Yu X.J., Yang M.J., Zhou B. (2015). Characterization of Somatic Mutations in Air Pollution-Related Lung Cancer. EBioMedicine.

[140] Yang L., Liu G., Li X. (2020). Small GTPase RAB6 deficiency promotes alveolar progenitor cell renewal and attenuates PM2.5-induced lung injury and fibrosis. Cell Death Dis..

[141] Li Y., Lin B., Hao D. (2023). Short-term PM(2.5) exposure induces transient lung injury and repair. J. Hazard. Mater..

[142] Ovrevik J., Refsnes M., Totlandsdal A.I. (2011). TACE/TGF-alpha/EGFR regulates CXCL8 in bronchial epithelial cells exposed to particulate matter components. Eur. Respir. J..

[143] Ning J., Pei Z., Wang M. (2023). Site-specific Atg13 methylation-mediated autophagy regulates epithelial inflammation in PM2.5-induced pulmonary fibrosis. J. Hazard. Mater..

[144] Shi Y., Zhao T., Yang X. (2019). PM(2.5)-induced alteration of DNA methylation and RNA-transcription are associated with inflammatory response and lung injury. Sci. Total Environ..

[145] Li X., Lv Y., Gao N. (2016). microRNA-802/Rnd3 pathway imposes on carcinogenesis and metastasis of fine particulate matter exposure. Oncotarget.

[146] Ning J., Li P., Zhang B. (2019). miRNAs deregulation in serum of mice is associated with lung cancer related pathway deregulation induced by PM2.5. Environ. Pollut..

[147] Fan R., Xu L., Cui B. (2023). Genomic Characterization Revealed PM(2.5)-Associated Mutational Signatures in Lung Cancer Including Activation of APOBEC3B. Environ. Sci. Technol..

[148] Jassim A., Rahrmann E.P., Simons B.D. (2023). Cancers make their own luck: theories of cancer origins. Nat. Rev. Cancer.

[149] Van Den Heuvel R., Den Hond E., Govarts E. (2016). Identification of PM10 characteristics involved in cellular responses in human bronchial epithelial cells (Beas-2B). Environ. Res..

[150] de Oliveira Alves N., Martins Pereira G., Di Domenico M. (2020). Inflammation response, oxidative stress and DNA damage caused by urban air pollution exposure increase in the lack of DNA repair XPC protein. Environ. Int..

[151] Xu J., Zhang Q., Su Z. (2022). Genetic damage and potential mechanism exploration under different air pollution patterns by multi-omics. Environ. Int..

[152] Xu J., Liu Y., Zhang Q. (2022). DNA damage, serum metabolomic alteration and carcinogenic risk associated with low-level air pollution. Environ. Pollut..

[153] Zhao L., Zhang M., Bai L. (2022). Real-world PM(2.5) exposure induces pathological injury and DNA damage associated with miRNAs and DNA methylation alteration in rat lungs. Environ. Sci. Pollut. Res. Int..

[154] Leclercq B., Kluza J., Antherieu S. (2018). Air pollution-derived PM(2.5) impairs mitochondrial function in healthy and chronic obstructive pulmonary diseased human bronchial epithelial cells. Environ. Pollut..

[155] Iwai K., Adachi S., Takahashi M. (2000). Early oxidative DNA damages and late development of lung cancer in diesel exhaust-exposed rats. Environ. Res..

[156] Abbas I., Verdin A., Escande F. (2016). In vitro short-term exposure to air pollution PM2.5-0.3 induced cell cycle alterations and genetic instability in a human lung cell coculture model. Environ. Res..

[157] Koh G., Degasperi A., Zou X. (2021). Mutational signatures: emerging concepts, caveats and clinical applications. Nat. Rev. Cancer.

[158] Chen Y.J., Roumeliotis T.I., Chang Y.H. (2020). Proteogenomics of Non-smoking Lung Cancer in East Asia Delineates Molecular Signatures of Pathogenesis and Progression. Cell.

[159] Zhang T., Joubert P., Ansari-Pour N. (2021). Genomic and evolutionary classification of lung cancer in never smokers. Nat. Genet..

[160] Espín-Pérez A., Krauskopf J., Chadeau-Hyam M. (2018). Short-term transcriptome and microRNAs responses to exposure to different air pollutants in two population studies. Environ. Pollut..

[161] Cayir A., Barrow T.M., Guo L. (2019). Exposure to environmental toxicants reduces global N6-methyladenosine RNA methylation and alters expression of RNA methylation modulator genes. Environ. Res..

[162] Yuan Q., Zhu H., Liu H. (2021). METTL3 regulates PM(2.5)-induced cell injury by targeting OSGIN1 in human airway epithelial cells. J. Hazard. Mater..

[163] Clifford R.L., Jones M.J., MacIsaac J.L. (2017). Inhalation of diesel exhaust and allergen alters human bronchial epithelium DNA methylation. J. Allergy Clin. Immunol..

[164] Jiang C.L., He S.W., Zhang Y.D. (2017). Air pollution and DNA methylation alterations in lung cancer: A systematic and comparative study. Oncotarget.

[165] Zheng L., Wang Y., Zhang Y. (2021). EGFR inhibitors regulate Ca(2+) concentration and apoptosis after PM(2.5) exposure based on a lung-mimic microfluidic system. Sci. Total Environ..

[166] Li M.Y., Liu L.Z., Li W. (2019). Ambient fine particulate matter inhibits 15-lipoxygenases to promote lung carcinogenesis. J. Exp. Clin. Cancer Res..

[167] Zheng L., Wang Y., Zhang Y. (2022). Investigation of PM(2.5)-induced carcinogenic effects through mediation of ErbB family based on DNA methylation and transcriptomics analysis by a lung-mimicking microfluidic platform. Ecotoxicol. Environ. Saf..

[168] Liu Y., Xu J., Shi J. (2023). Effects of short-term high-concentration exposure to PM(2.5) on pulmonary tissue damage and repair ability as well as innate immune events. Environ. Pollut..

[169] Chao X., Yi L., Lan L.L. (2020). Long-term PM(2.5) exposure increases the risk of non-small cell lung cancer (NSCLC) progression by enhancing interleukin-17a (IL-17a)-regulated proliferation and metastasis. Aging (Albany NY).

[170] Zhang J., Li X., Cheng W. (2022). Chronic carbon black nanoparticles exposure increases lung cancer risk by affecting the cell cycle via circulatory inflammation. Environ. Pollut..

[171] Lin C.M., Huang T.H., Chi M.C. (2022). N-acetylcysteine alleviates fine particulate matter (PM2.5)-induced lung injury by attenuation of ROS-mediated recruitment of neutrophils and Ly6C(high) monocytes and lung inflammation. Ecotoxicol. Environ. Saf..

[172] Liu J., Li S., Fei X. (2022). Increased alveolar epithelial TRAF6 via autophagy-dependent TRIM37 degradation mediates particulate matter-induced lung metastasis. Autophagy.

[173] Weng C.M., Lee M.J., He J.R. (2018). Diesel exhaust particles up-regulate interleukin-17A expression via ROS/NF-kappaB in airway epithelium. Biochem. Pharmacol..

[174] Yun Y., Gao R., Yue H. (2015). Synergistic effects of particulate matter (PM10) and SO2 on human non-small cell lung cancer A549 via ROS-mediated NF-kappaB activation. J. Environ. Sci..

[175] Xu F., Qiu X., Hu X. (2018). Effects on IL-1beta signaling activation induced by water and organic extracts of fine particulate matter (PM(2.5)) in vitro. Environ. Pollut..

[176] Bleck B., Tse D.B., Gordon T. (2010). Diesel exhaust particle-treated human bronchial epithelial cells upregulate Jagged-1 and OX40 ligand in myeloid dendritic cells via thymic stromal lymphopoietin. J. Immunol..

[177] Natarajan K., Gottipati K.R., Berhane K. (2016). Proteases and oxidant stress control organic dust induction of inflammatory gene expression in lung epithelial cells. Respir. Res..

[178] Chang C.Y., You R., Armstrong D. (2022). Chronic exposure to carbon black ultrafine particles reprograms macrophage metabolism and accelerates lung cancer. Sci. Adv..

[179] Ural B.B., Caron D.P., Dogra P. (2022). Inhaled particulate accumulation with age impairs immune function and architecture in human lung lymph nodes. Nat. Med..

[180] Zhang R., Chen S., Chen L. (2022). Single-cell transcriptomics reveals immune dysregulation mediated by IL-17A in initiation of chronic lung injuries upon real-ambient particulate matter exposure. Part. Fibre Toxicol..

[181] Zhu T., Li Y., Feng T. (2022). D-Limonene inhibits the occurrence and progression of LUAD through suppressing lipid droplet accumulation induced by PM(2.5) exposure in vivo and in vitro. Respir. Res..

[182] Fu Y., Lu R., Cui J. (2019). Inhibition of ATP citrate lyase (ACLY) protects airway epithelia from PM(2.5)-induced epithelial-mesenchymal transition. Ecotoxicol. Environ. Saf..

[183] Chen Q., Wang Y., Yang L. (2022). PM2.5 promotes NSCLC carcinogenesis through translationally and transcriptionally activating DLAT-mediated glycolysis reprograming. J. Exp. Clin. Cancer Res..

[184] Chen S., Li D., Zhang H. (2019). The development of a cell-based model for the assessment of carcinogenic potential upon long-term PM2.5 exposure. Environ. Int..

[185] Wang T.H., Huang K.Y., Chen C.C. (2023). PM2.5 promotes lung cancer progression through activation of the AhR-TMPRSS2-IL18 pathway. EMBO Mol. Med..

[186] Ferecatu I., Borot M.C., Bossard C. (2010). Polycyclic aromatic hydrocarbon components contribute to the mitochondria-antiapoptotic effect of fine particulate matter on human bronchial epithelial cells via the aryl hydrocarbon receptor. Part. Fibre Toxicol..

[187] Teranishi M., Toyooka T., Ohura T. (2010). Benzo[a]pyrene exposed to solar-simulated light inhibits apoptosis and augments carcinogenicity. Chem. Biol. Interact..

[188] Xu J., Huang L., Bao T. (2023). CircCDR1as mediates PM(2.5)-induced lung cancer progression by binding to SRSF1. Ecotoxicol. Environ. Saf..

[189] Reyes-Zárate E., Sánchez-Pérez Y., Gutiérrez-Ruiz M.C. (2016). Atmospheric particulate matter (PM10) exposure-induced cell cycle arrest and apoptosis evasion through STAT3 activation via PKCzeta and Src kinases in lung cells. Environ. Pollut..

[190] Xu H., Jiao X., Wu Y. (2019). Exosomes derived from PM2.5-treated lung cancer cells promote the growth of lung cancer via the Wnt3a/beta-catenin pathway. Oncol. Rep..

[191] Wei H., Liang F., Cheng W. (2017). The mechanisms for lung cancer risk of PM(2.5) : Induction of epithelial-mesenchymal transition and cancer stem cell properties in human non-small cell lung cancer cells. Environ. Toxicol..

[192] Jiang P., Hao S., Xie L. (2021). LncRNA NEAT1 contributes to the acquisition of a tumor like-phenotype induced by PM 2.5 in lung bronchial epithelial cells via HIF-1alpha activation. Environ. Sci. Pollut. Res. Int..

[193] Wang Y., Zhong Y., Hou T. (2019). PM2.5 induces EMT and promotes CSC properties by activating Notch pathway in vivo and vitro. Ecotoxicol. Environ. Saf..

[194] Ji S.M., Choi J.S., Lee J.Y. (2023). Mild exposure to fine particulate matter promotes angiogenesis in non-small cell lung carcinoma. Environ. Pollut..

[195] Zheng L., Liu S., Zhuang G. (2017). Signal Transductions of BEAS-2B Cells in Response to Carcinogenic PM(2.5) Exposure Based on a Microfluidic System. Anal. Chem..

[196] Xu X., Wang H., Liu S. (2016). TP53-dependent autophagy links the ATR-CHEK1 axis activation to proinflammatory VEGFA production in human bronchial epithelial cells exposed to fine particulate matter (PM2.5). Autophagy.

[197] Wang Y., Zhong Y., Zhang C. (2020). PM2.5 downregulates MicroRNA-139-5p and induces EMT in Bronchiolar Epithelium Cells by targeting Notch1. J. Cancer.

[198] Yang D., Ma M., Zhou W. (2017). Inhibition of miR-32 activity promoted EMT induced by PM2.5 exposure through the modulation of the Smad1-mediated signaling pathways in lung cancer cells. Chemosphere.

[199] Yang M., Ju L., Li C. (2022). MiR-582-3p participates in the regulation of biological behaviors of A549 cells by ambient PM(2.5) exposure. Environ. Sci. Pollut. Res. Int..

[200] Luo F., Wei H., Guo H. (2019). LncRNA MALAT1, an lncRNA acting via the miR-204/ZEB1 pathway, mediates the EMT induced by organic extract of PM(2.5) in lung bronchial epithelial cells. Am. J. Physiol. Lung Cell. Mol. Physiol..

[201] Deng X., Feng N., Zheng M. (2017). PM(2.5) exposure-induced autophagy is mediated by lncRNA loc146880 which also promotes the migration and invasion of lung cancer cells. Biochim. Biophys. Acta Gen. Subj..

[202] Wang Y., Zhang Y., Li Y. (2022). Mechanisms of Biochanin A Alleviating PM2.5 Organic Extracts-Induced EMT of A549 Cells through the PI3K/Akt Pathway. J. Nat. Prod..

[203] Gao R., Yun Y., Cai Z. (2019). PM(2.5)-associated nitro-PAH exposure promotes tumor cell metastasis through Hippo-YAP mediated transcriptional regulation. Sci. Total Environ..

[204] Liu L.Z., Wang M., Xin Q. (2020). The permissive role of TCTP in PM(2.5)/NNK-induced epithelial-mesenchymal transition in lung cells. J. Transl. Med..

[205] Fu Y., Li B., Yun J. (2021). lncRNA SOX2-OT ceRNA network enhances the malignancy of long-term PM(2.5)-exposed human bronchial epithelia. Ecotoxicol. Environ. Saf..

[206] Huang Y., Huang Y., Wang H. (2022). The effect of low molecular weight-polycyclic aromatic hydrocarbons responsive hsa_circ_0039929/hsa-miR-15b-3p_R-1/FGF2 circuit on inflammatory response of A549 cells via the PI3K/AKT pathway and epithelial-mesenchymal transition process. Environ. Toxicol..

[207] Park S.H., Yoon S.J., Choi S. (2022). Particulate matter promotes cancer metastasis through increased HBEGF expression in macrophages. Exp. Mol. Med..

[208] Olesiejuk K., Chałubiński M. (2023). How does particulate air pollution affect barrier functions and inflammatory activity of lung vascular endothelium?. Allergy.

[209] Soca-Chafre G., Avila-Vásquez H., Rueda-Romero C. (2021). Airborne particulate matter upregulates expression of early and late adhesion molecules and their receptors in a lung adenocarcinoma cell line. Environ. Res..

[210] Gao R., Sang N. (2020). Quasi-ultrafine particles promote cell metastasis via HMGB1-mediated cancer cell adhesion. Environ. Pollut..

[211] Wang Z., Wu Y., Pei C. (2022). Astragaloside IV pre-treatment attenuates PM2.5-induced lung injury in rats: Impact on autophagy, apoptosis and inflammation. Phytomedicine.

[212] Lin H.W., Shen T.J., Yang N.C. (2022). Luteolin Reduces Aqueous Extract PM2.5-induced Metastatic Activity in H460 Lung Cancer Cells. Int. J. Med. Sci..

[213] Pan J., Xue Y., Li S. (2022). PM(2.5) induces the distant metastasis of lung adenocarcinoma via promoting the stem cell properties of cancer cells. Environ. Pollut..

[214] Rocks N., Vanwinge C., Radermecker C. (2019). Ozone-primed neutrophils promote early steps of tumour cell metastasis to lungs by enhancing their NET production. Thorax.

[215] Fowler J.C., Jones P.H. (2022). Somatic Mutation: What Shapes the Mutational Landscape of Normal Epithelia?. Cancer Discov..

[216] Kakiuchi N., Ogawa S. (2021). Clonal expansion in non-cancer tissues. Nat. Rev. Cancer.

[217] Ferone G., Lee M.C., Sage J. (2020). Cells of origin of lung cancers: lessons from mouse studies. Genes Dev..

[218] Sikkema L., Ramírez-Suástegui C., Strobl D.C. (2023). An integrated cell atlas of the lung in health and disease. Nat. Med..

[219] Mainardi S., Mijimolle N., Francoz S. (2014). Identification of cancer initiating cells in K-Ras driven lung adenocarcinoma. Proc. Natl. Acad. Sci. USA.

[220] Chen Y., Toth R., Chocarro S. (2022). Club cells employ regeneration mechanisms during lung tumorigenesis. Nat. Commun..

[221] Traversi D., Cervella P., Gilli G. (2015). Evaluating the genotoxicity of urban PM2.5 using PCR-based methods in human lung cells and the Salmonella TA98 reverse test. Environ. Sci. Pollut. Res. Int..

[222] Quezada-Maldonado E.M., Cerrato-Izaguirre D., Morales-Bárcenas R. (2024). Mutational landscape induced by chronic exposure to environmental PM(10) and PM(2.5) in A549 lung epithelial cell. Chemosphere.

[223] Mo Y., Zhang Y., Zhang Y. (2021). Nickel nanoparticle-induced cell transformation: involvement of DNA damage and DNA repair defect through HIF-1alpha/miR-210/Rad52 pathway. J. Nanobiotechnol..

[224] Han B., Pei Z., Shi L. (2020). TiO(2) Nanoparticles Caused DNA Damage in Lung and Extra-Pulmonary Organs Through ROS-Activated FOXO3a Signaling Pathway After Intratracheal Administration in Rats. Int. J. Nanomed..

[225] Bhargava A., Tamrakar S., Aglawe A. (2018). Ultrafine particulate matter impairs mitochondrial redox homeostasis and activates phosphatidylinositol 3-kinase mediated DNA damage responses in lymphocytes. Environ. Pollut..

[226] Santibáñez-Andrade M., Sánchez-Pérez Y., Chirino Y.I. (2022). Particulate matter (PM(10)) destabilizes mitotic spindle through downregulation of SETD2 in A549 lung cancer cells. Chemosphere.

[227] Jamal-Hanjani M., Wilson G.A., McGranahan N. (2017). Tracking the Evolution of Non-Small-Cell Lung Cancer. N. Engl. J. Med..

[228] Butler K., Banday A.R. (2023). APOBEC3-mediated mutagenesis in cancer: causes, clinical significance and therapeutic potential. J. Hematol. Oncol..

[229] LoPiccolo J., Gusev A., Christiani D.C. (2024). Lung cancer in patients who have never smoked - an emerging disease. Nat. Rev. Clin. Oncol..

[230] Murphy C., Pandya T., Swanton C. (2025). Lung Cancer in Nonsmoking Individuals: A Review. JAMA.

[231] Blechter B., Cardenas A., Shi J. (2023). Household air pollution and epigenetic aging in Xuanwei, China. Environ. Int..

[232] Maghbooli Z., Hossein-Nezhad A., Adabi E. (2018). Air pollution during pregnancy and placental adaptation in the levels of global DNA methylation. PLoS One.

[233] Goodrich J.M., Reddy P., Naidoo R.N. (2016). Prenatal exposures and DNA methylation in newborns: a pilot study in Durban, South Africa. Environ. Sci. Process. Impacts.

[234] Capelo-Diz A., Lachiondo-Ortega S., Fernández-Ramos D. (2023). Hepatic levels of S-adenosylmethionine regulate the adaptive response to fasting. Cell Metab..

[235] Wu H., Eckhardt C.M., Baccarelli A.A. (2023). Molecular mechanisms of environmental exposures and human disease. Nat. Rev. Genet..

[236] Leclercq B., Platel A., Antherieu S. (2017). Genetic and epigenetic alterations in normal and sensitive COPD-diseased human bronchial epithelial cells repeatedly exposed to air pollution-derived PM(2.5). Environ. Pollut..

[237] Greten F.R., Grivennikov S.I. (2019). Inflammation and Cancer: Triggers, Mechanisms, and Consequences. Immunity.

[238] Wang W., Lin Y., Yang H. (2022). Internal Exposure and Distribution of Airborne Fine Particles in the Human Body: Methodology, Current Understandings, and Research Needs. Environ. Sci. Technol..

[239] Noël A., Xiao R., Perveen Z. (2016). Incomplete lung recovery following sub-acute inhalation of combustion-derived ultrafine particles in mice. Part. Fibre Toxicol..

[240] Niechoda A., Roslan M., Milewska K. (2024). Signalling Pathways of Inflammation and Cancer in Human Mononuclear Cells: Effect of Nanoparticle Air Pollutants. Cells.

[241] Curtius K., Wright N.A., Graham T.A. (2018). An evolutionary perspective on field cancerization. Nat. Rev. Cancer.

[242] Sabharwal S.S., Schumacker P.T. (2014). Mitochondrial ROS in cancer: initiators, amplifiers or an Achilles' heel?. Nat. Rev. Cancer.

[243] de Visser K.E., Joyce J.A. (2023). The evolving tumor microenvironment: From cancer initiation to metastatic outgrowth. Cancer Cell.

[244] Ma W., Xu L., Sun X. (2023). Using a human bronchial epithelial cell-based malignant transformation model to explore the function of hsa-miR-200 family in the progress of PM(2.5)-induced lung cancer development. Environ. Pollut..

[245] Bian W., Yu H., Zhang X. (2024). Particulate matters 2.5 induce tumor progression in lung cancer by increasing the activity of hnRNPA2B1 resulting in retarding mRNA decay of oxidative phosphorylation. IUBMB Life.

[246] Li Y., Tang D., Zhang J. (2023). LncRNA SPRY4-IT1 regulates 16HBE cell malignant transformation induced by particulate matter through DUSP6-ERK1/2-Chk1 signaling pathway. Chemosphere.

[247] You M., Xie Z., Zhang N. (2023). Signaling pathways in cancer metabolism: mechanisms and therapeutic targets. Signal Transduct. Targeted Ther..

[248] Pyambri M., Lacorte S., Jaumot J. (2023). Effects of Indoor Dust Exposure on Lung Cells: Association of Chemical Composition with Phenotypic and Lipid Changes in a 3D Lung Cancer Cell Model. Environ. Sci. Technol..

[249] Balla T. (2013). Phosphoinositides: tiny lipids with giant impact on cell regulation. Physiol. Rev..

[250] Wang Y., Wen Y., Chen Q. (2024). Downregulation of tRNA methyltransferase FTSJ1 by PM2.5 promotes glycolysis and malignancy of NSCLC via facilitating PGK1 expression and translation. Cell Death Dis..

[251] Jiang Y.J., Ho T.L., Chao C.C. (2024). Particulate matter facilitates amphiregulin-dependent lung cancer proliferation through glutamine metabolism. Int. J. Biol. Sci..

[252] Brabletz T., Jung A., Spaderna S. (2005). Opinion: migrating cancer stem cells - an integrated concept of malignant tumour progression. Nat. Rev. Cancer.

[253] Bisht A., Dey S., Kulshreshtha R. (2024). Integrated meta-analyses of genome-wide effects of PM(2.5) in human cells identifies widespread dysregulation of genes and pathways associated with cancer progression and patient survival. Sci. Total Environ..

[254] Li R., Yang L., Jiang N. (2020). Activated macrophages are crucial during acute PM2.5 exposure-induced angiogenesis in lung cancer. Oncol. Lett..

[255] You R., Lu W., Shan M. (2015). Nanoparticulate carbon black in cigarette smoke induces DNA cleavage and Th17-mediated emphysema. eLife.

[256] Lu W., You R., Yuan X. (2015). The microRNA miR-22 inhibits the histone deacetylase HDAC4 to promote T(H)17 cell-dependent emphysema. Nat. Immunol..

[257] Wang Z., Zhai Z., Chen C. (2022). Air pollution particles hijack peroxidasin to disrupt immunosurveillance and promote lung cancer. eLife.

[258] Gerstberger S., Jiang Q., Ganesh K. (2023). Metastasis. Cell.

[259] Qi H., Liu Y., Wang N. (2021). Lentinan Attenuated the PM2.5 Exposure-Induced Inflammatory Response, Epithelial-Mesenchymal Transition and Migration by Inhibiting the PVT1/miR-199a-5p/caveolin1 Pathway in Lung Cancer. DNA Cell Biol..

[260] Zheng L., Yang Z., Xue Z. (2024). Air-Liquid Interface Microfluidic Monitoring Sensor Platform for Studying Autophagy Regulation after PM2.5 Exposure. ACS Sens..

[261] Ji S., Guo Y., Ding J. (2024). Nontargeted Identification of Organic Components in Fine Particulate Matter Related to Lung Tumor Metastasis Based on an Adverse Outcome Pathway Strategy. Environ. Sci. Technol..

[262] Kanikarla-Marie P., Lam M., Menter D.G. (2017). Platelets, circulating tumor cells, and the circulome. Cancer Metastasis Rev..

[263] Al Bakir M., Huebner A., Martínez-Ruiz C. (2023). The evolution of non-small cell lung cancer metastases in TRACERx. Nature.

[264] Martínez-Ruiz C., Black J.R.M., Puttick C. (2023). Genomic-transcriptomic evolution in lung cancer and metastasis. Nature.

[265] Subramanian J., Velcheti V., Gao F. (2007). Presentation and stage-specific outcomes of lifelong never-smokers with non-small cell lung cancer (NSCLC). J. Thorac. Oncol..

[266] Jin Y., Yang F., Chen K. (2023). An overview of current development and barriers on liquid biopsy in patients with early-stage non-small-cell Lung cancer. Holist. Integr. Oncol..

[267] Yin Z., Su M., Li X. (2009). ERCC2, ERCC1 polymorphisms and haplotypes, cooking oil fume and lung adenocarcinoma risk in Chinese non-smoking females. J. Exp. Clin. Cancer Res..

[268] Chen K.C., Tsai S.W., Shie R.H. (2022). Indoor Air Pollution Increases the Risk of Lung Cancer. Int. J. Environ. Res. Publ. Health.

[269] Curbani F., de Oliveira Busato F., do Nascimento M.M. (2019). Inhale, exhale: Why particulate matter exposure in animal models are so acute?. Environ. Pollut..

[270] Maier K.L., Alessandrini F., Beck-Speier I. (2008). Health effects of ambient particulate matter--biological mechanisms and inflammatory responses to in vitro and in vivo particle exposures. Inhal. Toxicol..

[271] Loret T., Peyret E., Dubreuil M. (2016). Air-liquid interface exposure to aerosols of poorly soluble nanomaterials induces different biological activation levels compared to exposure to suspensions. Part. Fibre Toxicol..

[272] Xiao Y., Li Y., Jing X. (2025). Organoid models in oncology: advancing precision cancer therapy and vaccine development. Cancer Biol. Med..

[273] Li Z., Chen L., Wu J. (2025). A review of 3D bioprinting for organoids. Med. Rev..

[274] Becker S., Dailey L.A., Soukup J.M. (2005). Seasonal variations in air pollution particle-induced inflammatory mediator release and oxidative stress. Environ. Health Perspect..

[275] Villasclaras P., Jaén C., van Drooge B.L. (2022). Phenotypic and Metabolomic Characterization of 3D Lung Cell Cultures Exposed to Airborne Particulate Matter from Three Air Quality Network Stations in Catalonia. Toxics.

[276] Stark R., Grzelak M., Hadfield J. (2019). RNA sequencing: the teenage years. Nat. Rev. Genet..

[277] Unsihuay D., Mesa Sanchez D., Laskin J. (2021). Quantitative Mass Spectrometry Imaging of Biological Systems. Annu. Rev. Phys. Chem..

[278] Nunes J.B., Ijsselsteijn M.E., Abdelaal T. (2024). Integration of mass cytometry and mass spectrometry imaging for spatially resolved single-cell metabolic profiling. Nat. Methods.

[279] Bressan D., Battistoni G., Hannon G.J. (2023). The dawn of spatial omics. Science.

[280] Galassi C., Chan T.A., Vitale I. (2024). The hallmarks of cancer immune evasion. Cancer Cell.

[281] Yin L. (2025). High-throughput sequencing unveils tumor-immune interactions: From genomic alterations to clinical translation. Glob. Transl. Med..

[282] Liu Y., Sinjab A., Min J. (2025). Conserved spatial subtypes and cellular neighborhoods of cancer-associated fibroblasts revealed by single-cell spatial multi-omics. Cancer Cell.

[283] Liu Z., Liu W., Wei H. (2025). Elevated lactate production exacerbates PM2.5-induced pulmonary fibrosis by stabilizing TGF-beta1. J. Adv. Res..

[284] Shon J.C., Lee S.M., Jung J.H. (2020). Integrated metabolomics and lipidomics reveals high accumulation of polyunsaturated lysoglycerophospholipids in human lung fibroblasts exposed to fine particulate matter. Ecotoxicol. Environ. Saf..

[285] Vella G., Hua Y., Bergers G. (2023). High endothelial venules in cancer: Regulation, function, and therapeutic implication. Cancer Cell.

[286] Kim R., Hashimoto A., Markosyan N. (2022). Ferroptosis of tumour neutrophils causes immune suppression in cancer. Nature.

[287] Wang Y., Duan H., Zhang J. (2023). YAP1 protects against PM2.5-induced lung toxicity by suppressing pyroptosis and ferroptosis. Ecotoxicol. Environ. Saf..

[288] Yan K., Hou T., Zhu L. (2022). PM2.5 inhibits system Xc- activity to induce ferroptosis by activating the AMPK-Beclin1 pathway in acute lung injury. Ecotoxicol. Environ. Saf..

[289] Zhang Q., Wang W., Niu Y. (2019). The effects of fine particulate matter constituents on exhaled nitric oxide and DNA methylation in the arginase-nitric oxide synthase pathway. Environ. Int..

[290] Du X., Zhang Q., Jiang Y. (2022). Dynamic molecular choreography induced by traffic exposure: A randomized, crossover trial using multi-omics profiling. J. Hazard. Mater..

[291] Zhang Y., Chu M., Zhang J. (2019). Urine metabolites associated with cardiovascular effects from exposure of size-fractioned particulate matter in a subway environment: A randomized crossover study. Environ. Int..

[292] Mu L., Niu Z., Blair R.H. (2019). Metabolomics Profiling before, during, and after the Beijing Olympics: A Panel Study of Within-Individual Differences during Periods of High and Low Air Pollution. Environ. Health Perspect..

[293] Li J., Wang T., Wang Y. (2020). Particulate matter air pollution and the expression of microRNAs and pro-inflammatory genes: Association and mediation among children in Jinan, China. J. Hazard. Mater..

[294] Feng D., Cao K., He Z.Z. (2021). Short-Term Effects of Particle Sizes and Constituents on Blood Biomarkers among Healthy Young Adults in Guangzhou, China. Environ. Sci. Technol..

[295] Wang L., Cheng H., Wang D. (2019). Airway microbiome is associated with respiratory functions and responses to ambient particulate matter exposure. Ecotoxicol. Environ. Saf..

[296] Chen C., Li H., Niu Y. (2019). Impact of short-term exposure to fine particulate matter air pollution on urinary metabolome: A randomized, double-blind, crossover trial. Environ. Int..

[297] Wang M., Zhao J., Wang Y. (2020). Genome-wide DNA methylation analysis reveals significant impact of long-term ambient air pollution exposure on biological functions related to mitochondria and immune response. Environ. Pollut..

[298] Zhao Y., Xue L., Chen Q. (2020). Cardiorespiratory responses to fine particles during ambient PM(2.5) pollution waves: Findings from a randomized crossover trial in young healthy adults. Environ. Int..

[299] Liu B., Jiang M., Wu Y. (2025). Impact of air pollution on the progress-free survival of non-small cell lung cancer patients with anti-PD-1/PD-L1 immunotherapy: A cohort study. Environ. Pollut..

[300] Roy A., Chen J., Zhang X. (2023). A general framework for powerful confounder adjustment in omics association studies. Bioinformatics.

[301] Goh W.W.B., Wong L. (2018). Dealing with Confounders in Omics Analysis. Trends Biotechnol..

[302] Han S.S., Chow E., Ten Haaf K. (2020). Disparities of National Lung Cancer Screening Guidelines in the US Population. J. Natl. Cancer Inst..

[303] Krist A.H., Davidson K.W., US Preventive Services Task Force (2021). Screening for Lung Cancer: US Preventive Services Task Force Recommendation Statement. JAMA.

[304] Yang D., Liu Y., Bai C. (2020). Epidemiology of lung cancer and lung cancer screening programs in China and the United States. Cancer Lett..

[305] Cheng E.S., Weber M.F., Steinberg J. (2022). Evaluating risk factors for lung cancer among never-smoking individuals using two Australian studies. J. Cancer Res. Clin. Oncol..

[306] Marshall H.M., Fong K.M. (2024). Lung cancer screening - Time for an update?. Lung Cancer.

[307] Turner M.C., Jerrett M., Pope C.A. (2016). Long-Term Ozone Exposure and Mortality in a Large Prospective Study. Am. J. Respir. Crit. Care Med..

[308] Chen J., Atkinson R.W., Andersen Z.J. (2024). Long-term exposure to ambient air pollution and risk of lung cancer - A comparative analysis of incidence and mortality in four administrative cohorts in the ELAPSE study. Environ. Res..

[309] Ou Y., West J.J., Smith S.J. (2020). Air pollution control strategies directly limiting national health damages in the US. Nat. Commun..

[310] Chen Z.Y., Petetin H., Méndez Turrubiates R.F. (2024). Population exposure to multiple air pollutants and its compound episodes in Europe. Nat. Commun..

[311] Feng Y., Ning M., Lei Y. (2019). Defending blue sky in China: Effectiveness of the "Air Pollution Prevention and Control Action Plan" on air quality improvements from 2013 to 2017. J. Environ. Manag..

[312] Xu M., Qin Z., Zhang S. (2021). Integrated assessment of cleaning air policy in China: A case study for Beijing-Tianjin-Hebei region. J. Clean. Prod..

[313] Lu X., Zhang S., Xing J. (2020). Progress of Air Pollution Control in China and Its Challenges and Opportunities in the Ecological Civilization Era. Engineering.

[314] Daellenbach K.R., Uzu G., Jiang J. (2020). Sources of particulate-matter air pollution and its oxidative potential in Europe. Nature.

[315] Zhong J., Karlsson O., Wang G. (2017). B vitamins attenuate the epigenetic effects of ambient fine particles in a pilot human intervention trial. Proc. Natl. Acad. Sci. USA.

[316] Chen T., Song L., Zhong X. (2023). Dietary polyunsaturated fatty acids intake, air pollution, and the risk of lung cancer: A prospective study in UK biobank. Sci. Total Environ..

[317] Li H., Liu Q., Zou Z. (2021). L-arginine supplementation to mitigate cardiovascular effects of walking outside in the context of traffic-related air pollution in participants with elevated blood pressure: A randomized, double-blind, placebo-controlled trial. Environ. Int..

