# Supplementary material for: Hallmarks of lung cancer driven by inhalable particulate matter: From cell-intrinsic oncogenic traits to microenvironment remodeling
Source: Innovation (Camb). 2026 Mar 18;7(8):101347. doi: 10.1016/j.xinn.2026.101347 (PMC13457187; doi:10.1016/j.xinn.2026.101347)
Supplement: Supplementary file 1 [file mmc1.pdf]

The Innovation, Volume *7*

Supplemental Information

Hallmarks of lung cancer driven by inhalable particulate matter: From cell-intrinsic oncogenic traits to microenvironment remodeling

Xiaoqiu Yuan, Xinrui Li, Chen Wang, Emilia Lim, Yunchu Wei, Ruoyi Jin, Lin Weng, Takahiro Karasaki, Hao Luo, Li Qing, Zhiwei Zhou, Qisi Jiang, Olga Chervova, Fan Yang, Fang Wu, Kai Zhang, Cheng-Yen Chang, Farrah Kheradmand, Jianpeng Sheng, David Carbone, Jun Wang, and Kezhong Chen

Supplementary materials

Figure S1 Global distribution of PM2.5 levels (1990-2000) and lung cancer research relevant to PM 2

Figure S2 Global distribution of PM2.5 levels (2001-2010) and lung cancer research relevant to PM 3

Figure S3 Global distribution of PM2.5 levels (2011-2020) and lung cancer research relevant to PM 4

Figure S4 Classical oncogenic pathways activated by PM 5

Table S1 Epidemiological studies of PM levels and lung cancer risk 6

Table S2 Initiating cells of lung cancer 6

Table S3 PM-mediated mechanisms of lung cancer promotion 7

Figure S1 Global distribution of PM2.5 levels (1990-2000) and lung cancer research relevant to PM

lat: latitude, lon: longitude.

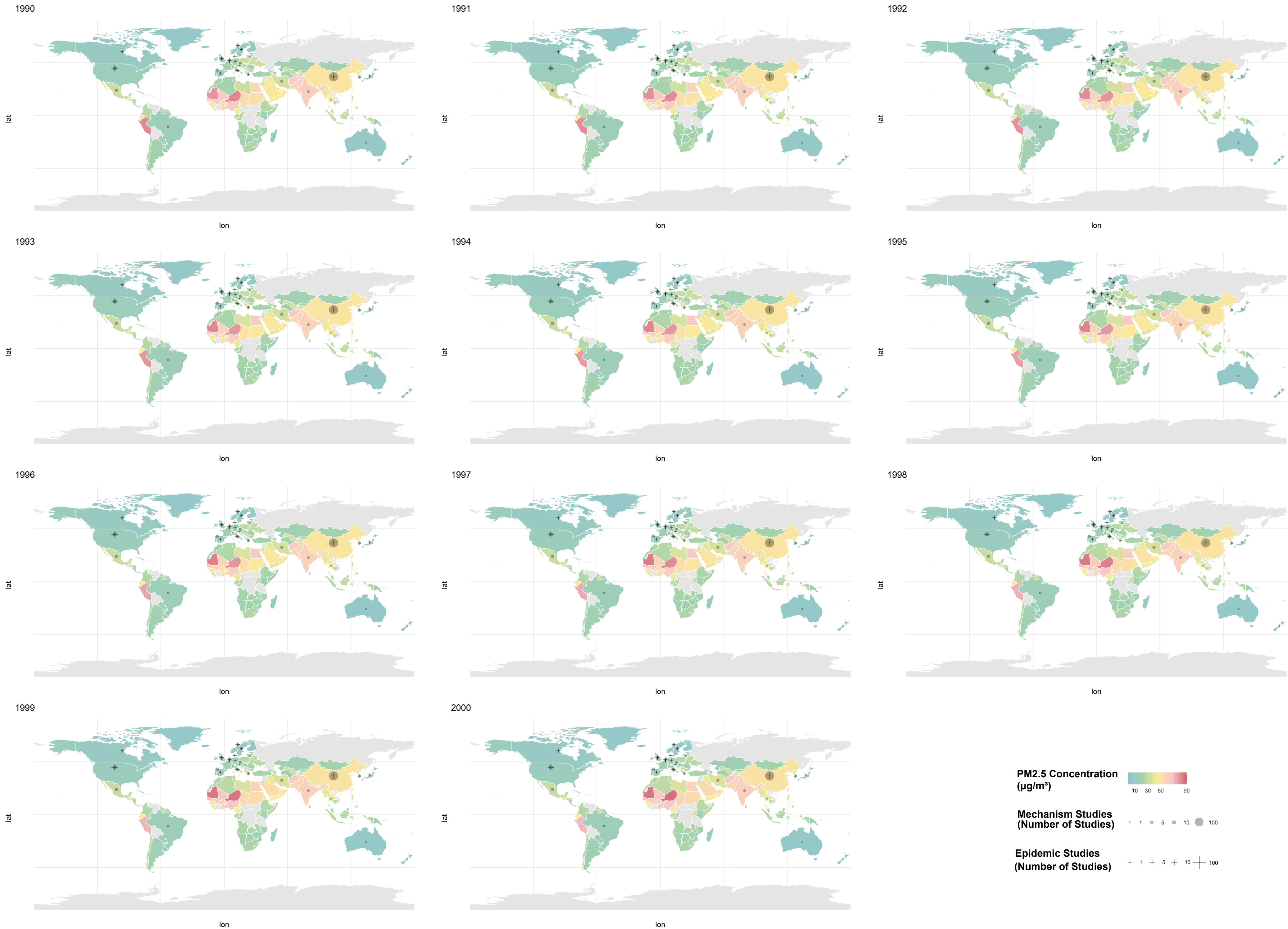

Figure S2 Global distribution of PM2.5 levels (2001-2010) and lung cancer research relevant to PM

lat: latitude, lon: longitude.

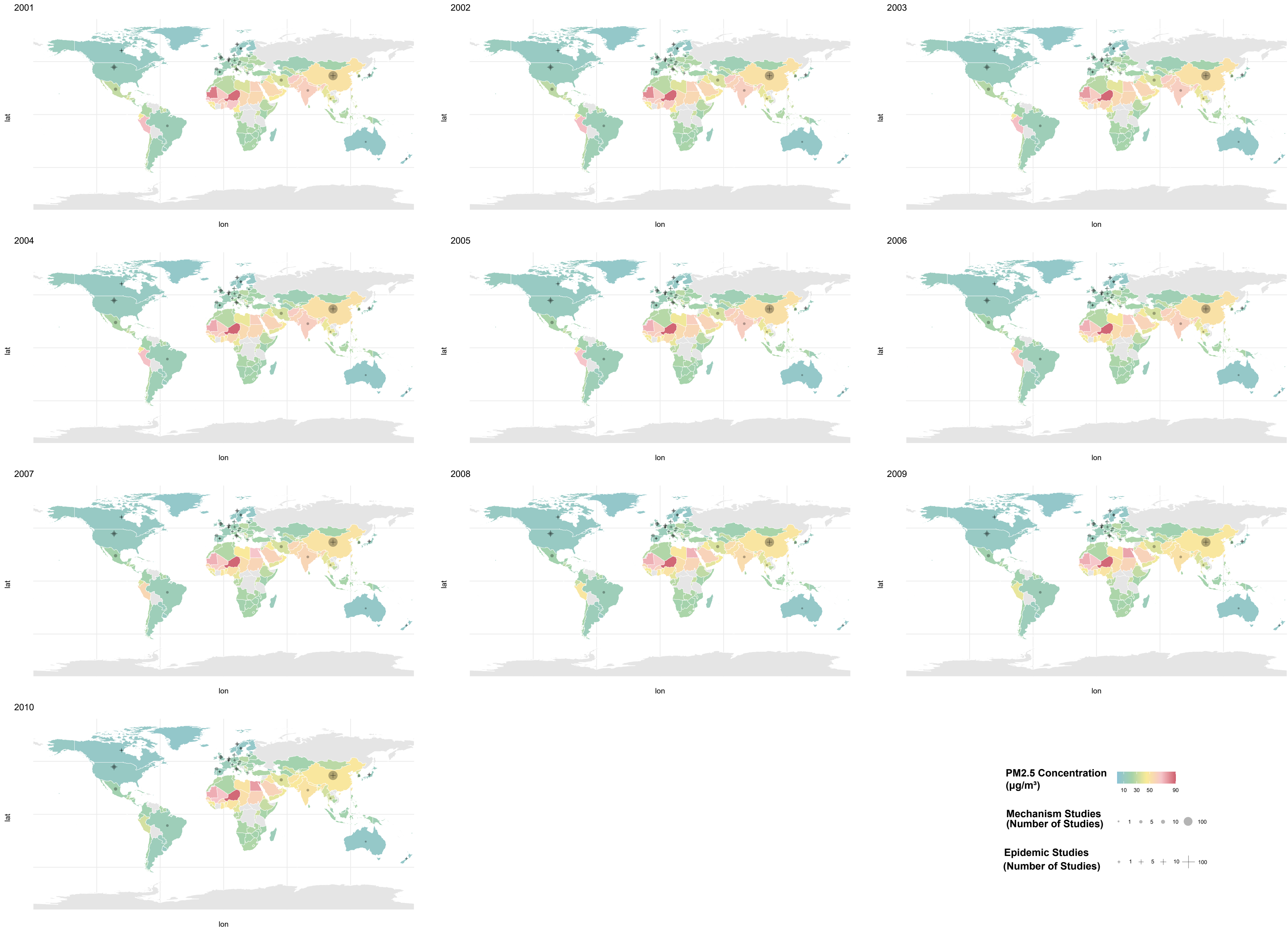

Figure S3 Global distribution of PM2.5 levels (2011-2020) and lung cancer research relevant to PM

lat: latitude, lon: longitude.

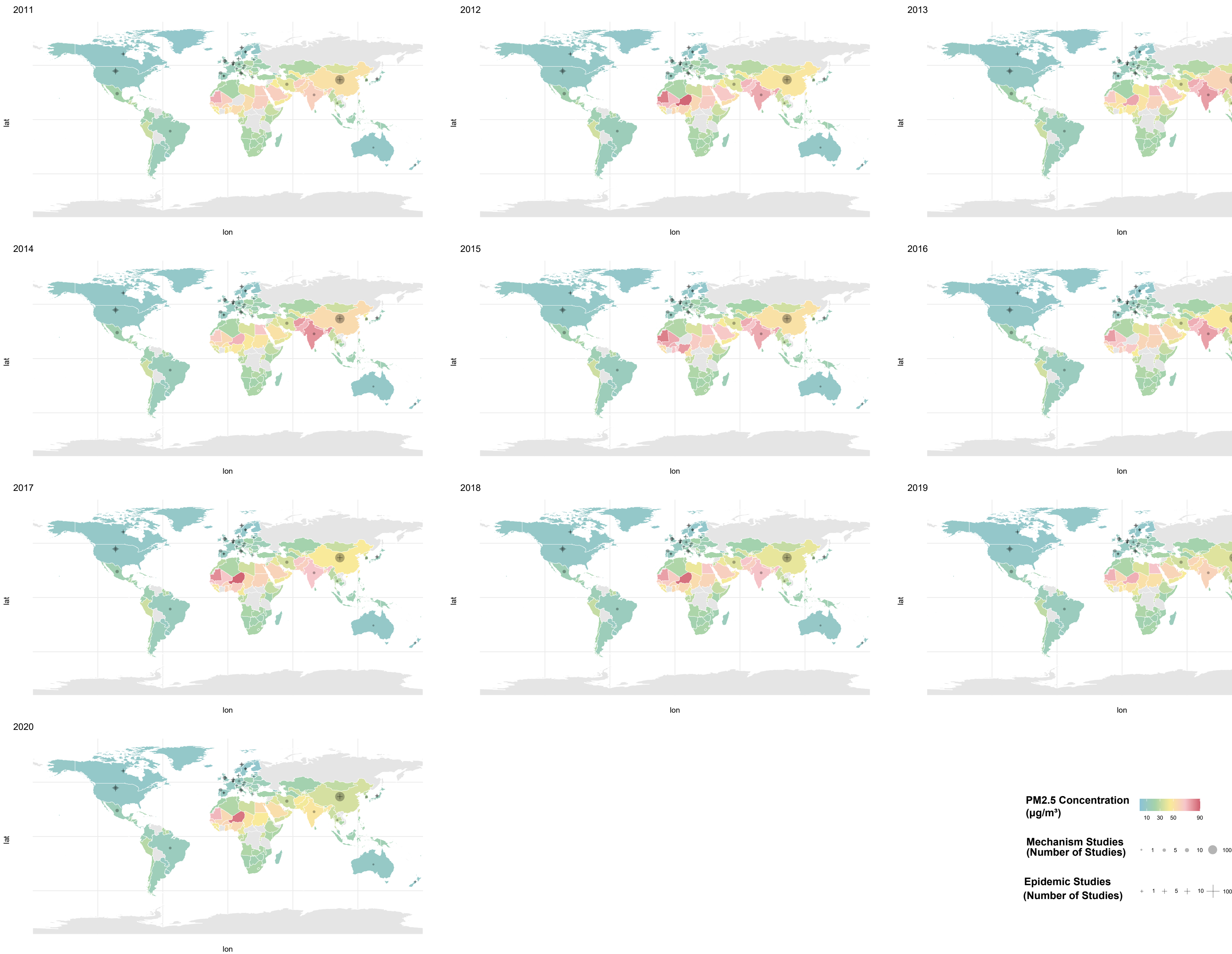

Figure S4 Classical oncogenic pathways activated by PM

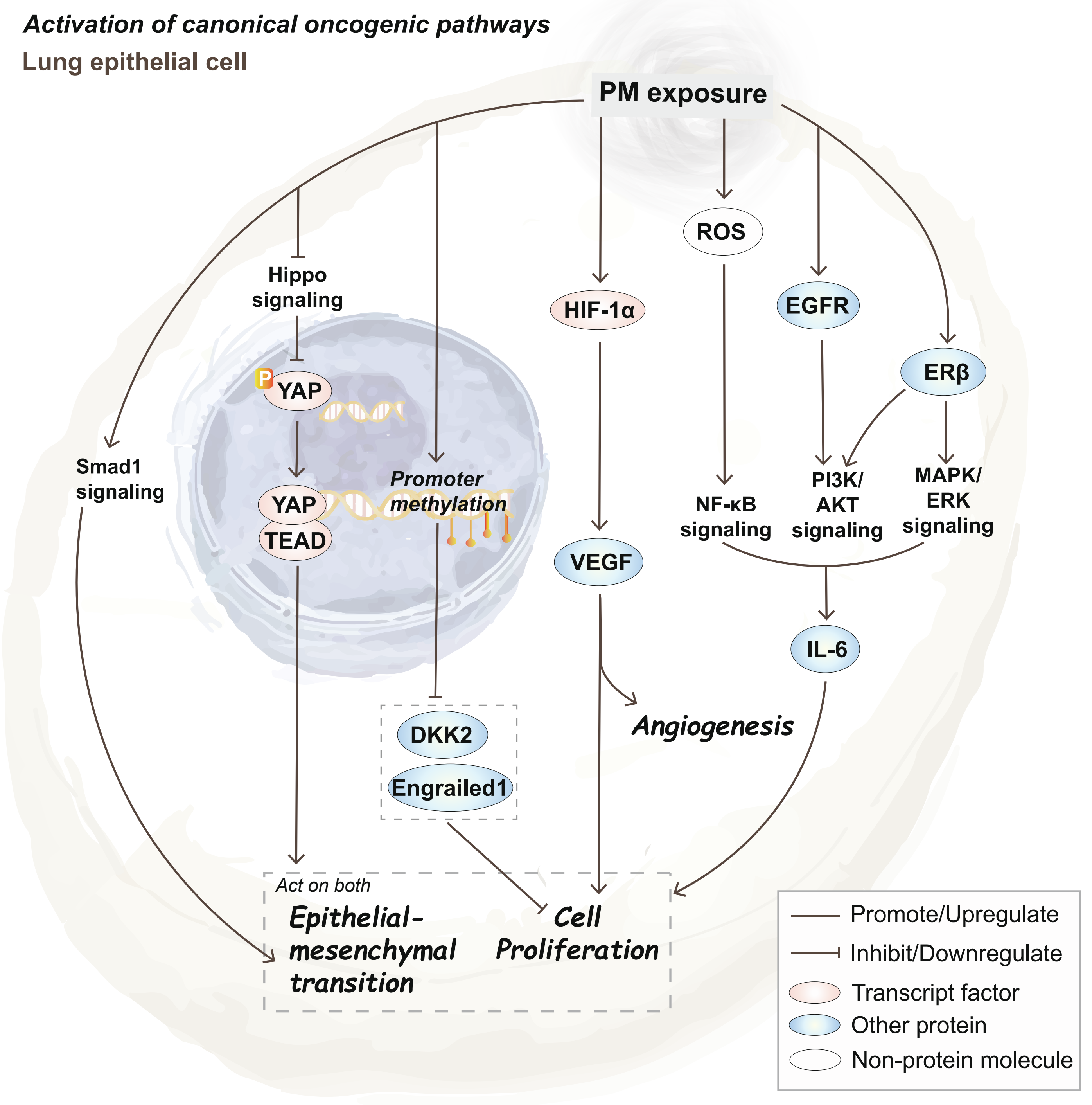

Table S1 Epidemiological studies of PM levels and lung cancer risk

See the affiliated excel file for this table.

Table S2 Initiating cells of lung cancer

Initiating cell lineage	Genetic alterations		Risk factor exposure	Sample/Tumor incidence /MPLC/latency	Histology and Location	Note
**AT1 cell**[1]	$Kras^{G12D}$		NA	NA/NA/NA/ 6months	lepidic adenocarcinoma	KRAS(G12D) in differentiated AT1 cells reprograms them slowly and asynchronously back into AT2 stem cells
**AT2 cell**[2]	$Kras^{G12D}$	$Trp53^{+/+}$	NA	8/100% /100%/ 10weeks	LUAD/NA	p53 promotes AT1 differentiation
		$Trp53^{fl/fl}$		5/100%/100%/ 10weeks		NA
**AT2 cell**[3]	$EGFR^{L858R}$		PBS	9/33.3%/22.2%/ 10weeks	LUAD/NA	NA
			5μg PM, three times per week for 3 weeks	9/77.8%/44.4%/ 10weeks		PM-mediated AT2 cell reprogramming
			50μg PM, three times per week for 3 weeks	9/77.8%/66.6%/ 10weeks		
**AT2 cell**[4]	Ptenf/f; Cdkn2abf/f	SOX2	NA	6/100% /NA/7-8 months	LSCC /peripheral	SOX2 is the determining oncogenic switch in promoting LSCC
**Basal cell**[4]				4/100% /NA/7-8 months		

**Club cell**[4]				11/73%/NA/ 7-9 months	LSCC/central	
		Fgfr1		19/53%/NA/ 10-15months	LADC and sarcoma/central	
**Club cell**[5]	$Kras^{G12V}$	$Trp53^{fl/fl}$	NA	5/100%/100%/ 4months	LUAD/ distal bronchioles	the progressive loss of CC10 and the increasing gain of SPC; late onset of Sox2
**BASC**[6]	Gprc5a-KO		two-week i.p. injections of NNK (100 mg/kg of body weight)	8/100%/NA/ 10months	LUAD/NA	EGFR and NF-kB signaling are dramatically upregulated
**PNEC**[7]	$Trp53^{fl/fl}$ $Rb1^{fl/fl}$		NA	30/83.3%/NA/ 362 days (median latency)	SCLC	Neuroendocrine cells are the predominant cells of origin of SCLC

AT1/2 cell: Alveolar Type 1/2 cell; BASC: Bronchioalveolar Stem Cell. PNEC: Pulmonary Neuroendocrine Cell. LUAD: Lung Adenocarcinoma; LSCC: Lung Squamous Cell Carcinoma. LADC: Lung Adenosquamous Carcinoma; SCLC: Small Cell Lung Cancer. NNK: tobacco carcinogen. MLC: Multiple lung cancers.

Table S3 PM-mediated mechanisms of lung cancer promotion

See the affiliated excel file for this table.

References

1. Juul, N. H. *et al.* KRAS(G12D) drives lepidic adenocarcinoma through stem-cell reprogramming. *Nature* 619, 860–867 (2023).
2. Kaiser, A. M. *et al.* p53 governs an AT1 differentiation programme in lung cancer suppression. *Nature* 619, 851–859 (2023).
3. Hill, W. *et al.* Lung adenocarcinoma promotion by air pollutants. *Nature* 616, 159–167 (2023).
4. Ferone, G. *et al.* SOX2 Is the Determining Oncogenic Switch in Promoting Lung Squamous Cell Carcinoma from Different Cells of Origin. *Cancer Cell* 30, 519–532 (2016).
5. Rosigkeit, S. *et al.* Definitive evidence for Club cells as progenitors for mutant Kras/Trp53-deficient lung cancer. *Int J Cancer* 149, 1670–1682 (2021).
6. Yin, H. *et al.* Identification of Active Bronchioalveolar Stem Cells as the Cell of Origin in Lung Adenocarcinoma. *Cancer Res* 82, 1025–1037 (2022).
7. Sutherland, K. D. *et al.* Cell of origin of small cell lung cancer: Inactivation of Trp53 and Rb1 in distinct cell types of adult mouse lung. *Cancer Cell* 19, 754–764 (2011).
